# Characterization of novel glycosyl hydrolases discovered by cell wall glycan directed monoclonal antibody screening and metagenome analysis of maize aerial root mucilage

**DOI:** 10.1371/journal.pone.0204525

**Published:** 2018-09-26

**Authors:** Tania Pozzo, Shawn M. Higdon, Sivakumar Pattathil, Michael G. Hahn, Alan B. Bennett

**Affiliations:** 1 Department of Plant Sciences, University of California, Davis, CA, United States of America; 2 Mascoma LLC (Lallemand Inc.), Lebanon, NH, United States of America; 3 BioEnergy Science Center (BESC), Oak Ridge National Laboratory, Oak Ridge, Tennessee, United States of America; 4 Complex Carbohydrate Research Center, University of Georgia, Athens, GA, United States of America; Iowa State University, UNITED STATES

## Abstract

An indigenous maize landrace from the Sierra Mixe region of Oaxaca, Mexico exhibits extensive formation of aerial roots which exude large volumes of a polysaccharide-rich gel matrix or “mucilage” that harbors diazotrophic microbiota. We hypothesize that the mucilage associated microbial community carries out multiple functions, including disassembly of the mucilage polysaccharide. *In situ*, hydrolytic assay of the mucilage revealed endogenous arabinofuranosidase, galactosidase, fucosidase, mannosidase and xylanase activities. Screening the mucilage against plant cell wall glycan-specific monoclonal antibodies recognized the presence of carbohydrate epitopes of hemicellulosic polysaccharides like xyloglucan (both non-fucosylated and fucosylated), xylan (both substituted and unsubstituted xylan domains) and pectic-arabinogalactans, all of which are potential carbon sources for mucilage microbial residents. Mucilage metagenome annotation using MG-RAST identified the members forming the microbial community, and gene fragments with predicted functions associated with carbohydrate disassembly. Data from the *in situ* hydrolytic activity and monoclonal antibody screening assays were used to guide the selection of five full length genes with predicted glycosyl hydrolase function from the GenBank database that were similar to gene fragments of high relative abundance in the mucilage metagenomes. These five genes were then synthesized for recombinant production in *Escherichia coli*. Here we report the characterization of an α-N-arabinofuranosidase (GH51) and an oligosaccharide reducing-end xylanase (GH8) from *Flavobacterium johnsoniae;* an α-L-fucosidase (GH29) and a xylan β-1,4 xylosidase (GH39) from *Spirosoma linguale*, and a β-mannosidase (GH2) from *Agrobacterium fabrum*. Biochemical characterization of these enzymes revealed a β-Mannosidase that also exhibits a secondary activity towards the cleavage of galactosyl residues. We also describe two xylanases (GH8 and GH39) from underexplored glycosyl hydrolase families, one thermostable α-L-Fucosidase (GH29) and a thermostable α-N-Arabinofuranosidase (GH51).

## Introduction

The large increase in shotgun metagenomic sequence data from environmental samples collected around the world provides extensive information regarding the taxonomic distribution of microbial communities. The sequence information contained within these metagenomes also serves as a potential resource for the discovery of novel enzymatic machinery, which can be achieved by establishing links between a given environmental sequencing data set and the metabolic processes that confer functions of interest within the targeted community [1–3]. Additionally, environmental sequencing studies also enable researchers to streamline the development of biocatalyst pipelines in a more efficient manner. Conducting stand-alone enzyme screening assays in high throughput, automated formats for a desired functionality is likely to be inefficient when compared to more targeted approaches that utilize environmental sequence analysis at the community-level to extract specific protein coding sequences from a well-characterized environment [4–6].

The application of functional metagenomics targeting the investigation of bacterial carbohydrate active enzymes (CAZymes) has recently emerged, where ecological changes that affect global carbon cycling in natural environments can now be monitored. This approach has provided unique opportunities to rapidly scan the microbial functionality of any ecosystem for new pools of glycosyl hydrolase (GH) biodiversity that can be used to create biocatalysts for the improvement of biotechnological processes [7]. However, the proliferation and establishment of each species within the microbial community of a given environment will largely depend on a number of factors, including the presence of specific glyconutrients with high bioavailability [3, 8].

**Fig 1:**
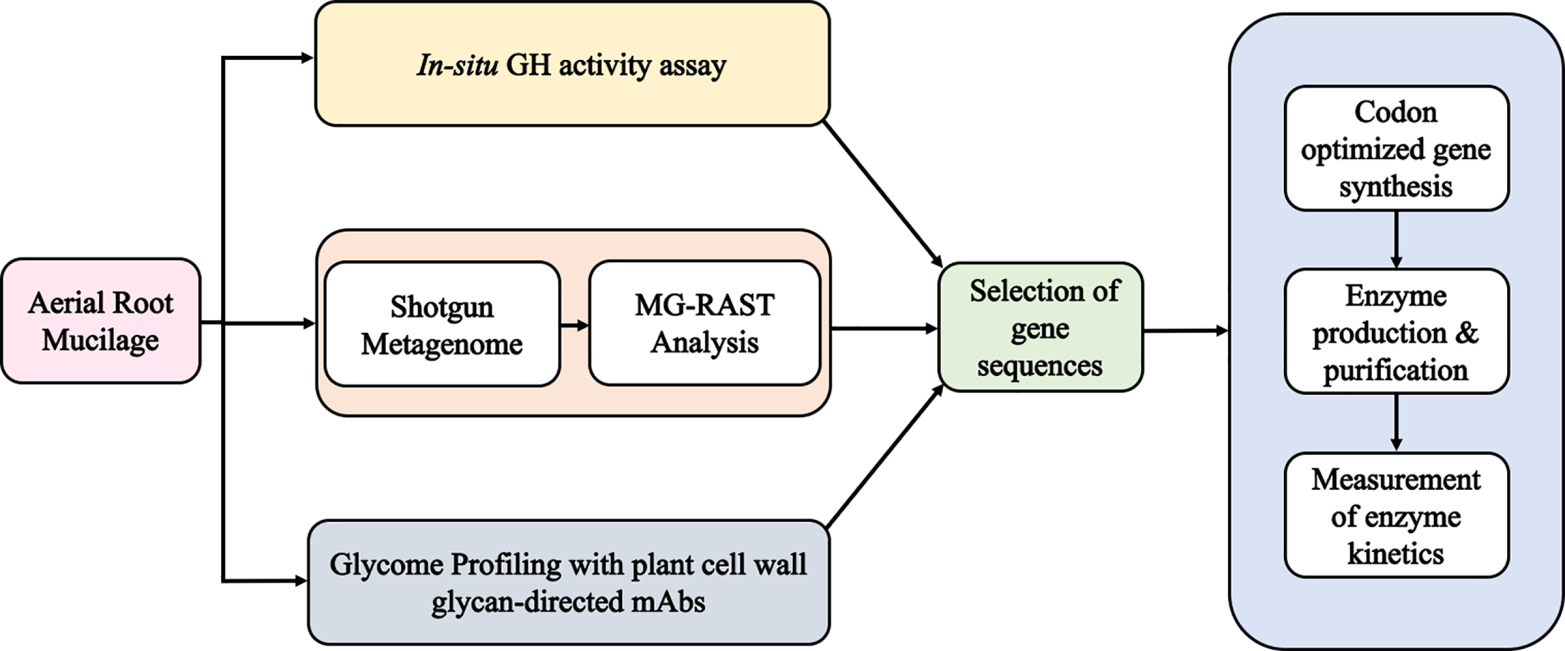
Pipeline for the discovery of novel glycosidic enzymes using aerial root mucilage. Aerial root mucilage samples were analyzed using three different approaches: an *in-situ* assay for endogenous glycosyl hydrolase (GH) activity; an enzyme linked immuno sorbent assay (ELISA) to detect non-cellulosic glycan epitopes using monoclonal antibodies (mAbs) and shotgun metagenomics. The metagenomes from five maize aerial root mucilage samples were analyzed using MG-RAST tools by querying all of the metagenome sequences against the SEED database. This enabled the identification of partial sequences from the mucilage metagenome samples that had high relative abundance and high sequence similarity to full length GenBank coding sequences for genes encoding GH activities. Results from the *in-situ* GH activity and ELISA assays of Sierra Mixe mucilage revealed GH activities that were likely to be relevant in mucilage polysaccharide disassembly. These data were then used to guide the selection of five full length GH encoding gene sequences with the targeted functional predictions, which were then subjected to artificial gene synthesis, recombinant production and biochemical characterization.

Discovery of GHs with new substrate specificities from metagenome environments that are rich in non-cellulosic sugar linkages, or with unique linkages, are extremely valuable for sustainable technologies that utilize biomass conversion of non-cellulosic polymers contained within plant-based feedstocks such as tailored prebiotic fibers from bio-refineries [9, 10]. In this study, a unique variety of maize from the Sierra Mixe region of Oaxaca, Mexico was selected as the candidate source for enzyme discovery based on its observed development of an elaborate aerial root network that extensively secretes a carbohydrate-rich gel matrix or “mucilage”[11]. Preliminary *in-situ* assays for endogenous GH activities within the aerial root exudate suggested that the mucilage environment harbored CAZymes that act upon arabinosyl, galactosyl, fucosyl, mannosyl and xylosyl sugar residues derived from mucilage glycans. Furthermore, using an enzyme-linked immunosorbent assay (ELISA) to monitor plant-cell wall glycan epitopes present in the secreted mucilage provided structural insights and corroborated the *in-situ* enzyme activities detected. Metagenomes from aerial root mucilage were found to harbor a microbiome with a high relative abundance of GH sequences, and the integration of all three data types guided our selection of five gene sequences from the mucilage metagenomes that exhibited high sequence similarity to GH sequences within the GenBank and Carbohydrate-Active enZYmes (CAZy) databases [12, 13]. Here we report the gene synthesis, recombinant production and biochemical characterization of five GHs, and collectively, the results validate the strategy of combining glycome profiling, environmental sequencing, genome analysis, and synthetic biology to elucidate the functional characteristics of novel subgroups of enzymes (GHs, other CAZymes, or any other enzymes) of specific relevance to ecosystems of interest (Fig 1).

## Material and methods

### Chemicals

4-Nitrophenyl (4NP), 4-Nitrophenyl (4NP)-β-D-glucopyranoside (4NP-β-Glc), 4NP-β-D-xylopyranoside (4NP-β-Xyl), 4NP-β-D-galactopyranoside (4NP-β-Gal), 4NP-β-D-manopyranoside (4NP-β-Man), 4NP-α-L-fucopyranoside (4NP-α-Fuc), 4NP-β-D-xylopyranoside (4NP-β-Xyl), 4NP-α-D-mannopyranoside (4NP-α-Man) and 4NP-α-L-arabinopyranoside (4NP-α-Ara) were purchased from Sigma-Aldrich (Steinheim, Germany). 4NP-β-xylotrioside (4NP-β-(Xyl)_3_) was purchased from Megazyme (Wicklow, Ireland).

### *In situ* glycosyl hydrolase activities endogenous to the aerial root mucilage

To assay mucilage for endogenous GH activities, a high throughput colorimetric assay utilizing 4-Nitrophenyl (4NP)-conjugated glycosides was implemented in 96 well format similar to previously reported methods for determining extracellular enzyme activity in environmental samples [14]. Carrying out this assay required large volumes of fresh mucilage samples, which were generated by collecting mucilage from greenhouse-grown Sierra Mixe maize plants at U.C. Davis. The mechanism of detection for this assay relied on quantification of 4NP molecules that were liberated from their respective 4NP-conjugated glycosides following incubation with freshly collected mucilage at 28 °C. This assay was conducted as a time course study over 48 hours of continuous monitoring with 30-minute intervals between absorbance measurements at a wavelength of 400 nm using the BioTek Synergy^™^ Mx microplate reader. Quantification of 4NP levels within each mucilage-incubated sample was achieved by interpretation from a linear standard curve that was generated by measuring the absorbance values of 4NP dissolved in fresh mucilage over a range of concentrations (0.02–1 mM). To prepare the standard curve wells, stock solutions were prepared by dissolving the respective amounts of 4NP with Molecular Biology Grade water. Next, 100 μL of each stock solution was combined with 150 μL of freshly collected mucilage such that the final concentration of 4NP in each well reflected a value within the range of the standard. For each 4NP substrate (4NP-β-Glc, 4NP-β-Xyl, 4NP-β-Gal, 4NP-β-Man, 4NP- α-Fuc, 4NP-α-Xyl, 4NP-α-Man, 4NP-α-Ara), 100 μL of a stock solution was combined with 150 μL of mucilage to achieve a final assay 4NP concentration of 4 mM. Each 4NP standard and sample was analyzed over 8 replications.

### Plant cell wall glycan directed monoclonal antibody screening of mucilage

Fresh aerial root mucilage samples collected off Sierra Mixe maize plants grown in greenhouses at U.C. Davis were utilized to generate aerial root mucilage samples that lacked low molecular weight sugar molecules. To achieve this, ethanol-insoluble mucilage samples (EIMS) were generated by precipitating the high molecular weight polysaccharide present in the fresh mucilage samples with high concentrations of absolute ethanol at a volumetric ratio of 3:1 (ethanol:mucilage) [15, 16]. The ethanol-soluble fraction of the solution was decanted, and the precipitate was re-constituted in Milli-Q water to generate the EIMS. These EIMS were then subjected to hydrolysis using trifluoroacetic acid (TFA) at different final concentrations (0.05 M, 0.2 M and 0.5 M) for 1 hour at 100 °C. Each hydrolysis reaction began with 3 mg of lyophilized mucilage that was re-constituted in 1 mL of sterile Milli-Q water. The hydrolysis reactions were initiated by adding appropriate volumes of 1 M TFA to achieve the desired final concentration and were subsequently terminated and neutralized through the dropwise addition of 1 M sodium hydroxide. The neutralized samples containing the long-chain oligosaccharides were then transferred to a 96-well plate and allowed to dry overnight at 37 °C. Next, the enzyme-linked immunosorbent assay (ELISA) for cell wall glycan-directed monoclonal antibody based screening was performed on each TFA-hydrolyzed mucilage sample [17]. ELISA screening of the mucilage preps was conducted using a comprehensive suite of plant cell wall glycan-directed monoclonal antibodies (mAbs) directed against diverse glycan epitopes dispersed among all major non-cellulosic glycans of plant cell walls except rhamnogalacturonan II [18]. These mAbs were obtained from laboratory stocks (CCRC, JIM and MAC series) at the Complex Carbohydrate Research Center (available through CarboSource Services; http://www.carbosource.net) or from BioSupplies (Australia) (BG1, LAMP). Additional information about the antibody library used for these experiments can be found at Wall*Mab*DB (www.wallmabdb.net).

### Aerial root mucilage metagenome analysis with MG-RAST

The five sequenced metagenomic libraries used in this study were generated as part of a related project examining the microbiomes associated with maize plants from the same geographic origin [11]. All five metagenomes were derived from mucilage samples that were collected from maize plants grown in the Sierra Mixe region of Mexico. Four of the samples were collected on August 1, 2008 and the fifth was collected on August 27^th^, 2013. Each collected sample was shipped to U.C. Davis, where total DNA was then extracted using a DNA isolation kit (Mo Bio Laboratories, Inc, USA). Illumina sequencing libraries were prepared using total DNA extracts from each mucilage sample with a procedure modified from the Nextera transposase-based library construction method and multiplex barcoding. All mucilage metagenome libraries were sequenced using the Illumina MiSeq and HiSeq 2000 instruments. Sequences were de-multiplexed and trimmed using Trimmomatic (version 0.33) with the following parameters: Illuminaclip 2:30:10, Headcrop:15, Leading:20, Trailing:20, Sliding window:4:20, and Minlen:100. PhiX and maize sequences (genomic, chloroplast and mitochondrial) within the mucilage metagenomes were screened for using Bowtie2 [19] against the PhiX genome (Genbank acc# NC_001422.1) and *Zea mays* cultivar B73 draft genome (RefSeq assembly acc# GCF_000005005.2). These sequences were then filtered out of the mucilage metagenomes that were uploaded to MG-RAST.

The trimmed and filtered sequence data for all five mucilage metagenomes were analyzed using MG-RAST web servers and exist as public records that were assigned the following IDs: samples from 2008 (mgm4504365.3, mgm4504364.3, mgm4504362.3 and mgm4504361.3) were uploaded to the server as fastq files containing the reads that survived the read processing described above, and the sample from 2013 (mgm4550815.3) was uploaded to the server as a list of contigs in fasta format that were assembled from the surviving fastq reads using the de Bruijn graph assembly program IDBA-UD v1.1.0 [20]. In terms of the total number of uploaded sequences for the five mucilage metagenomes on MG-RAST, mgm4504365.3 had 178,184 sequences, mgm4504364.3 had 19,282 sequences, mgm4504362.3 had 265,446 sequences, mgm4504361.3 had 5,024 sequences, and mgm4550815.3 had 74,429 sequences. Additionally, the average sequence length values for the mgm4504365.3, mgm4504364.3, mgm4504362.3, mgm4504361.3 and mgm4550815.3 metagenome libraries on MG-RAST were 105 ± 21 bp, 150 ± 21 bp, 109 ±25 bp, 149 ± 23 bp, and 1,475 ± 345 bp respectively. The reads from each aerial root mucilage metagenome library were analyzed collectively to evaluate the relative abundance of functional gene categories within the subsystems database using MG-RAST version 4.0.3. S1 Fig shows the workflow of the analysis made using MG-RAST. The overall microbial diversity within the aerial root mucilage samples was then assessed by querying the reads of all five metagenomes against the Refseq database using the analysis tool with the default settings, also using MG-RAST version 4.0.3 [21]. Results from the query of all reads against the Refseq database were then filtered based on phylum (S2 Fig, S1 Table) and class (S2 Table).

Using MG-RAST version 3.3.6, subsytems annotation of all aerial root mucilage metagenome samples was carried out using the default settings (maximum e-value cut off value of 1e^-5^, minimum percent identity cutoff value of 60, minimum alignment length cutoff value of 15 (S3 Fig. and S3 Table). The subsystem designated as “Carbohydrate” was evaluated in detail to identify bacterial genes that were predicted to encode CAZymes involved in mucilage polysaccharide catabolism. This process enabled the identification of partial DNA sequences from the mucilage metagenomes that were similar to known GenBank sequence annotations of previously reported bacterial genomes. The identified reference genome sequences were then utilized for artificial gene synthesis rather than using a PCR based approach to extract the actual enzyme coding sequences from the environmental samples because the original DNA extractions that were used to make the sequencing libraries were not available. These sequences of interest for CAZyme production are presented in Table 1, which provides relevant information regarding how the sequences were selected based on having a relatively high number of abundance hits across all five mucilage metagenome libraries, their alignment scores to genomic sequences in the GenBank database, the length of the reported sequence alignment, and the sequence alignment e-value scores.

**Table 1:** Glycosyl hydrolases selected for gene synthesis and metagenomic sequence query results. Analysis of the five mucilage metagenomes using the strategies depicted in Fig 1 and S1 Fig led to the selection of the following full-length coding sequences that were annotated in genomes that had been previously deposited to the GenBank database. “GH Family” corresponds to the selected enzyme coding sequence’s designation within the CAZy database. “Abundance Hits” refers to the number of partial sequences across all five mucilage metagenome samples that had sequence alignment hits to the selected full-length GH coding sequence. “Sequence Similarity” indicates the percentage of sequence similarity produced by alignment between the mucilage metagenome partial sequences and the full-length coding sequence from GenBank that was selected for gene synthesis.

Bacteria Name	Enzyme Name	Abundance Hits	Sequence Similarity (%)	E Value	Identity	UniProt ID	GH Family	MW kDa
*Flavobaterium* *Johnsoniae UW101*	α-N-Arabinofuranosidase (FjArf51)	273	94	4e-23	49/52	A5FF88	51	75
*Spirosoma* *Linguale* *DSM74*	α-L-Fucosidase (SlFuc29)	789	79	3e-83	136/172	D2QK56	29	55
*Agrobacterium fabrum str*. *C58*	β-Mannosidase (AfMan2)	1214	89	1e-133	213/238	Q7CZ23	2	93
*Spirosoma* *Linguale* *DSM74*	Xylan β-1,4 Xylosidase (SlXyn39)	1236	89	1e-135	217/245	D2QHX5	39	65
*Flavobaterium* *Johnsoniae UW101*	Oligosaccharide reducing end Xylanase (FjXyn8)	1010	81	1e-109	176/216	A5FD37	8	50

### Gene synthesis, recombinant protein production and purification

The five genes for which the gene products were predicted to have activities similar to those involved in mucilage polysaccharide catabolism that were selected for further characterization are listed in Table 1 with their corresponding Uniprot IDs [22]. The species origin and predicted function of each are as follows: α-L-fucosidase (D2QK56) and a xylan β-1,4 xylosidase (D2QHX5) from *Spirosoma linguale*, an α-N-arabinofuranosidase (A5FF88) and an oligosaccharide reducing-end xylanase (A5FD37) from *Flavobacterium johnsoniae*, and a β-mannosidase (Q7CZ23) from *Agrobacterium fabrum* str. C58. DNA sequences for these genes were codon optimized for recombinant production in *Escherichia coli* (*E*. *coli*) (S4 Table). Transformation constructs were generated by GenScript (Piscataway, NJ, USA) using chemically synthesized genes. The synthetic genes were cloned into the pET-28a (+) vector (Novagen) that contains a C-terminal, 6x poly-histidine tag. All constructs were transformed into *E*. *coli* BL21(DE3) pLysS competent cells (Novagen) by heat shock for protein production.

Small batch protein production and purification of each enzyme was carried out as described previously [23]. Briefly, the isopropyl β-D-1-thiogalactopyranoside (IPTG) concentration was optimized over a range from 0.1 to 0.5 mM for 50 mL cultures using Terrific Broth (TB). The incubation temperature was set to either 16 or 37 °C to improve the production of each soluble protein (if the production at 37 °C was low, the incubation temperature was lowered to 16 °C following the addition of IPTG) and the total protein from each was purified using the His-Spin Protein Miniprep kit from Zymo Research (Irvine, U.S.A). Confirmation of successful small-scale production was achieved by carrying out protein production in 200 mL of TB that was supplemented with 50 μg/mL kanamycin using a 1 L shake flask. Initial incubations were at 37 °C with shaking at 200 rpm. Induction of enzyme production was performed using the optimized amount of IPTG depending on the construct administered once the optical density of the liquid culture at λ = 600 nm reached a value of 0.6. Following chemical induction of protein production, cultivation times continued up to 16 hours with shaking at 200 rpm. The cell cultures were centrifuged (13,000 ×g, 10 min, 4 °C), and cell pellets were re-suspended in lysis buffer (20 mM imidazole, 20 mM Tris-HCl, 0.75 M NaCl, pH 7.5). Cell suspensions were lysed enzymatically as described by the European Molecular Biology Laboratory Protocol (www.embl.de), followed by treatment with Benzonase® Nuclease (EMD Millipore) for 30 minutes. Cell lysates were then immediately subjected to an additional round of centrifugation (30 min, 15,000 ×g, 4 °C). The His-tagged proteins were purified from the resulting supernatant using 1 mL HisTrap affinity columns (GE health care, Germany) that utilize a nickel sulfate solution to carry out immobilized metal ion affinity chromatography (IMAC). Fractions containing the purified enzyme were pooled and dialyzed against 50 mM citrate phosphate buffer over a range from pH 3 to pH 5 and phosphate buffer from pH 6 to pH8. The successful purification of each enzyme was confirmed by SDS-PAGE using a 12% Mini-PROTEAN TGX Precast Protein Gel and the Precision Plus Protein^™^ Kaleidoscope^™^ Standard from Bio-Rad Laboratories, Inc. (pipeline summarized with SDS-PAGE image in S4 Fig). Protein concentrations were quantified using the Micro BCA™ Protein Assay Kit from Thermo Scientific.

### Biochemical characterization of glycosyl hydrolases

Substrate specificity for each enzyme was determined by carrying out individual reactions with each of the following 4NP substrates: 4NP-β-Glc, 4NP-β-Xyl, 4NP-β-Gal, 4NP-β-Man, 4NP-α-Fuc, 4NP-α-Xyl, 4NP-α-Man, or 4NP-α-Ara. The optimal reaction conditions for hydrolysis of the 4NP-conjugated glycosides that corresponded to the specificity of each GH were determined by continuously monitoring sample absorbance at λ = 400 nm for a total duration of 60 minutes with 30 second intervals using different pH buffered solutions. Citrate-Phosphate buffer was used for reactions occurring over a pH range from 3.0 to 5.0 and Phosphate buffer was used for reactions occurring over a pH range from 6.0 to 8.0. Once the optimal pH was determined for each GH, reactions were carried out at different temperatures ranging from 30 °C to 60 °C to identify the optimal temperature for enzymatic hydrolysis. Standard curves were generated using 4NP for each combination of pH and temperature. Next, each 4NP substrate listed above was screened for hydrolytic activity against all five recombinant GH enzymes by adding the substrate to a final concentration of 1 mM, and the reactions were carried out under the optimal hydrolytic conditions for each enzyme over 50 minutes with 50 mM final buffer concentrations. If a secondary activity was detected for a recombinant GH protein was detected during the 4NP substrate screen, further characterization for the secondary activity was then carried out at the enzyme kinetic level. The kinetic parameters were obtained using the optimum pH at two temperatures using the software Graph Pad Prism6 (Graph Pad Software Inc., San Diego, CA) by fitting the rate of the reaction and substrate concentration to the Michaelis–Menten equation. All enzymatic reactions were run in triplicate.

### Phylogenetic analysis of glycosyl hydrolase family sequences

Multiple sequence alignment and gene tree analyses were carried out for each GH family that corresponded to the enzymes characterized in the present study. Amino acid sequences of bacterial enzymes from GH families 2, 8, 29, 39 and 51 were identified from the respective subsets of “Characterized” sequences within the CAZy database, where the subset of “Characterized” sequences refers to those that had been assigned enzyme class (EC) numbers based on experimental data according to rules set by the Nomenclature Committee of the International Union of Biochemistry and Molecular Biology (NC-IUBMB). These “Characterized” sequences from each GH family of interest were subsequently downloaded in FASTA format from the NCBI GenBank database (see S5 Table for sequence accessions used in the analysis). These sequences were then subjected to multiple sequence alignment and phylogenetic tree building using Geneious version 11.5. Multiple sequence alignments were carried out using ClustalW v 2.1 using the default settings of the Geneious module, and the trees were created using the UPGMA method with bootstrap resampling over 100 replications.

## Results and discussion

As an integral component of their development, plants generate root exudates that are primarily comprised of high molecular weight polysaccharide (mucilage) and specialized metabolites that serve as a means to facilitate information exchange with the root microbiome (rhizobiome) in order to meet demands imposed by their surrounding environment [24]. Because mucilage polysaccharides from the aerial roots of Sierra Mixe maize have the potential to be utilized as a carbon source by the resident microbial community, we sought to gather insight regarding the enzymatic machinery that is likely to be associated with polysaccharide disassembly within the aerial root mucilage environment.

### Detection of *in situ* glycosyl hydrolase activities endogenous to mucilage

**Fig 2:**
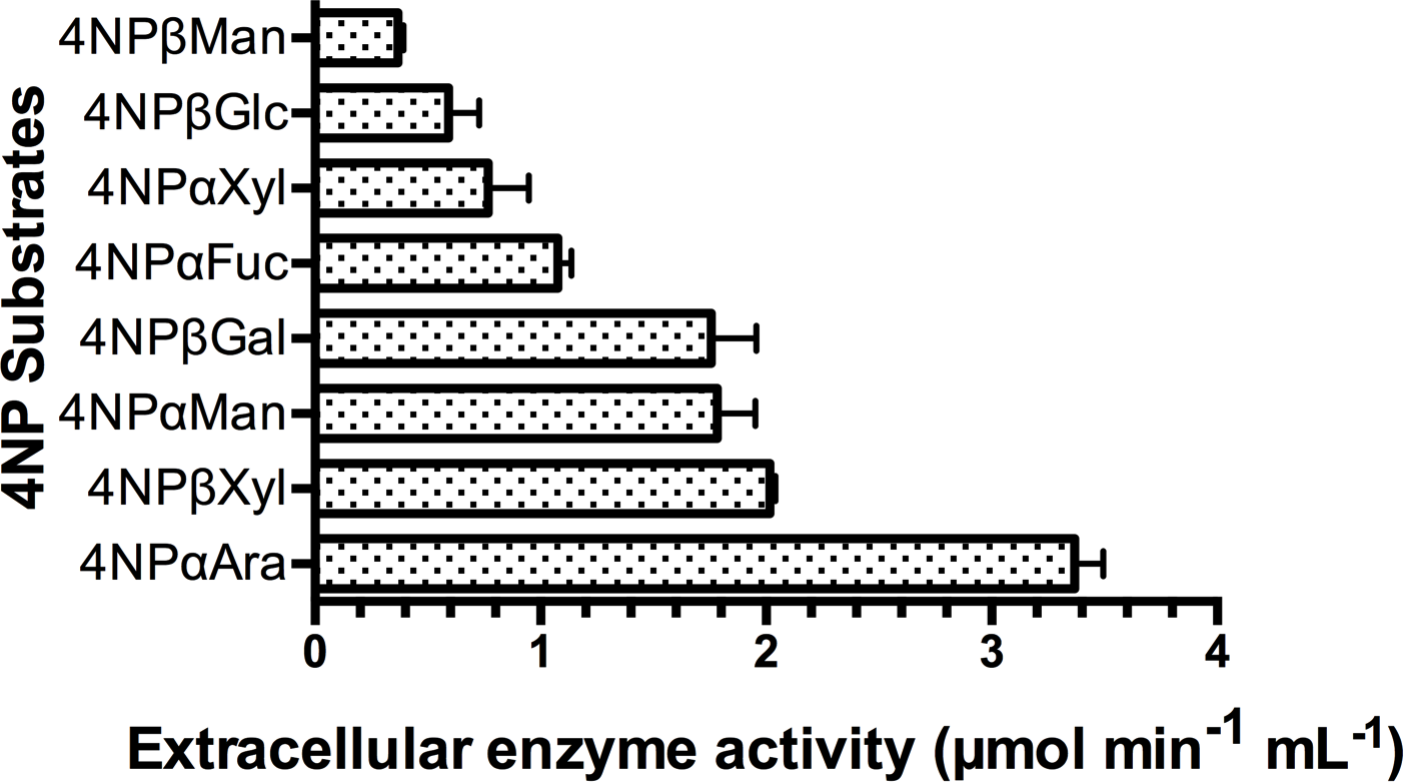
Detection and quantification of glycosyl hydrolase activity endogenous to aerial root mucilage. Freshly collected mucilage was spiked with different 4-Nitrophenyl Glycoside substrates to assess the hydrolytic activity of endogenous mucilage enzymes. 4NP liberation was monitored by spectrophotometry at λ = 400nm for 48 hours at 28°C.

The production of mucilage by the aerial roots is likely to be a dynamic process, where the polysaccharide structures present within the mucilage at any time represents a balance of newly secreted mucilage and the evolving structures of mucilage polysaccharides that are being metabolized by the diversity of mucilage-localized microbes. In an attempt to confirm the presence of enzymatic activity within the mucilage, 4NP-conjugated glycosidic substrates were spiked into freshly collected mucilage samples in order to monitor the GH activity of the mucilage *in situ*. While this colorimetric method allowed us to observe and quantify the presence of GH activity endogenous to the aerial root mucilage of Sierra Mixe maize, it also provided a direct line of evidence to support the presence of GH activities that target a diverse range of monosaccharide types within the mucilage (Fig 2).

The highest enzymatic activity observed within the mucilage was determined to be from enzymes acting on the 4NP-α-arabinofuranoside substrate, where these incubations exhibited the release of 4NP residues at a rate of 3.37 μmol•min^-1^•mL^-1^. Independent incubations of mucilage with 4NP-β-xylopyranoside, 4NP-α-mannopyranoside and 4NP-β-galactopyranoside substrates each demonstrated similar levels of 4NP liberation at 2.02, 1.78 and 1.76 μmol•min^-1^•mL^-1^ respectively. Endogenous enzymes that facilitated the liberation of 4NP residues from 4NP-α-fucopyranoside substrates showed only the fifth highest glycosidase activity rate (1.08 μmol•min^-1^•mL^-1^) in these assays. Enzymatic activities within the mucilage on 4NP-α-xylopyranoside, 4NP-β-glucopyranoside and 4NP-β-mannopyranoside substrates were determined to be the lowest among the eight substrates used in this set of assays (rates of 0.77, 0.59 and 0.37 μmol•min^-1^•mL^-1^). While the endogenous CAZyme activities detected within the mucilage cannot be concluded to be fully attributed to microbial CAZyme activities because of the possibility that plant-derived CAZymes may also populate the aerial root mucilage, this experiment was crucial for motivating further investigation because it revealed the presence of active enzymes likely to be involved in the catabolism of mucilage polysaccharide. These differences in substrate specificities indicated that the mucilage possesses a diverse range of GH activities and we can presume that relative levels of GH enzymes may be dynamic and vary over time, with our assays providing a snapshot in time.

### Mucilage polysaccharide structural insights

**Fig 3:**
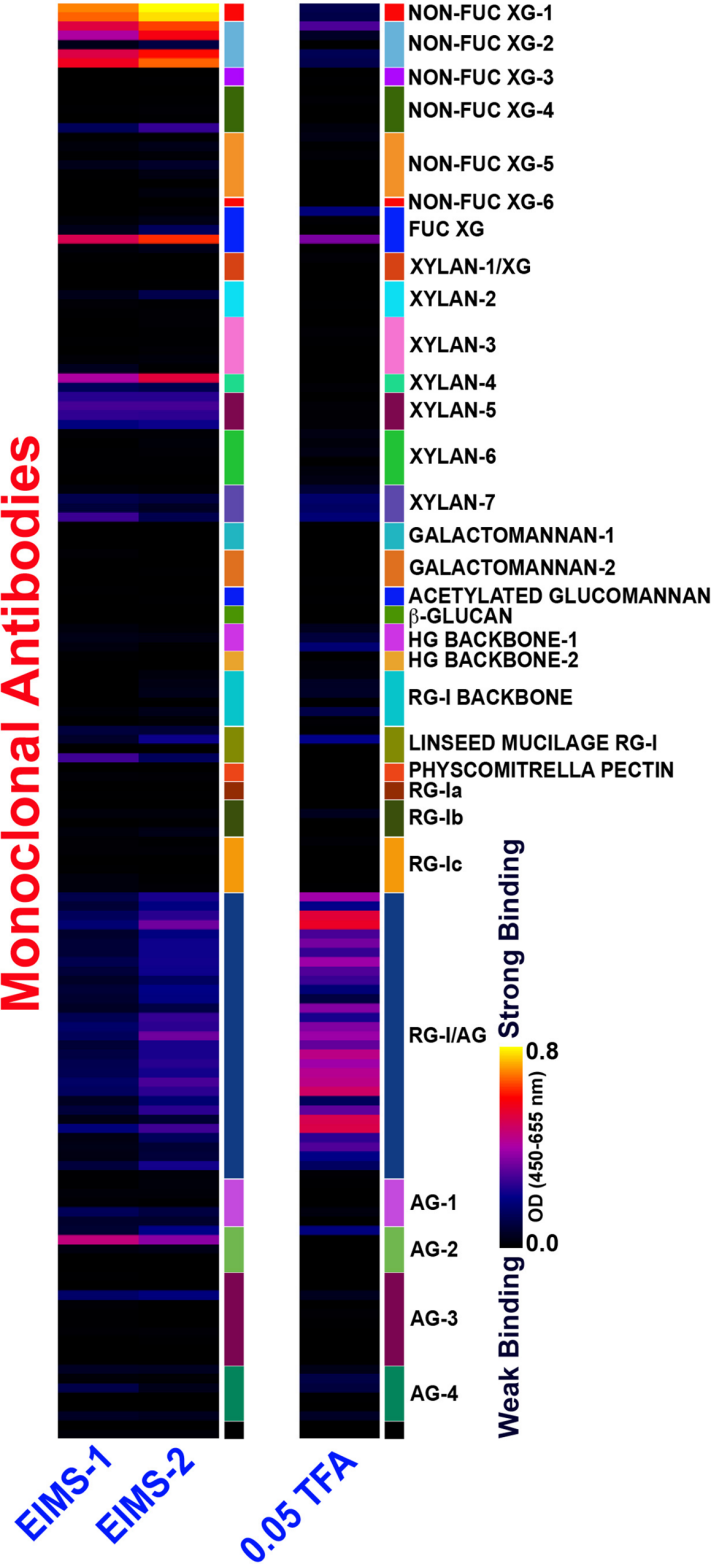
Screening of mucilage polysaccharide samples against cell wall glycan directed monoclonal antibodies. Untreated ethanol insoluble mucilage samples (EIMS) and ethanol insoluble mucilage samples that were pretreated with different concentrations of TFA (0.05 and 0.5 mM) were ELISA-screened against plant cell wall glycan-directed mAbs that recognize diverse epitopes in most major non-cellulosic glycans. The principal cell wall glycans recognized by each antibody clade are shown at the right, with the following abbreviations: XG xyloglucan, HG homogalacturonan, RG rhamnogalacturonan, and AG arabinogalactan. The color scheme of the heatmap depicts mAb binding strengths with dark blue representing no binding, red intermediate and bright yellow strongest binding.

A screening assay to detect non-cellulosic plant cell wall glycan epitopes using a suite of monoclonal antibodies (mAb) was conducted to characterize the structural features of the complex mucilage glycans. This screening assay confirmed that the glycan content within the mucilage is highly heterogeneous (Fig 3). The ethanol-insoluble mucilage samples (EIMS1 and EIMS2) exhibited binding to the following cell wall glycan-directed mAbs that recognize epitopes in hemicellulosic polysaccharides (xyloglucans, xylans): non-fucosylated xyloglucan-1 and -2 antibody clades, which bind to the L side-chain of xyloglucans (Tuomivaara et al., submitted for publication), a single mAb from the fucosylated xyloglucan clade (CCRC-M84) that is specific for difucosylated xyloglucan (nFFG) (Tuomivaara et al., submitted for publication); one antibody in xylan-4 (CCRC-M154) that is specific for arabino-substituted xylans [25], several antibodies specific for 4-O-methyl glucuronic acid side-chains on xylans (xylan-5 clade) [25], and a couple of antibodies from the xylan-7 clade, which bind unsubstituted xylan epitopes [25]. In addition, a number of antibodies in the RG-I/AG clade, which mostly recognize β-1,6 galactan epitopes with varying degrees of substitution [25] that are present on rhamnogalacturonan I (RG-I) (13) and arabinogalactan glycoproteins (AGPs) bind moderately.

Performing the same assay with mucilage samples that had been treated by mild acid hydrolysis (0.05M TFA, 100 °C) resulted in a different pattern of mAb binding. Most of the hemicellulosic (xyloglucan and xylan) epitopes were lost after the mild acid hydrolysis. Only weak binding by a couple xyloglucan-directed antibodies that bind to L-side-chain or the nFFG epitope remained. The binding of the xylan-directed antibodies was completely eliminated by the mild acid treatment, indicating that these epitopes were attached to the mucilage polymer by acid-labile linkages, perhaps in a configuration similar to that described for APAP1 [26]. In contrast, binding of antibodies in the RG-I/AG clade was increased in the 0.05M TFA hydrolyzed mucilage sample, probably because acid-labile substituents on the galactans (e.g., arabinosyl residues) were removed by the acid treatment thereby uncovering the underlying galactan epitopes recognized by these antibodies. Stronger (0.5 M) TFA hydrolysis treatment resulted in the almost complete removal of all antibody binding to the mucilage except for a couple of RG-I/AG antibodies, probably due to extensive hydrolysis and fragmentation of the mucilage polymer and hence loss of epitopes in the stronger acid environment (Fig 3). Collectively, the top five most abundant GH activities detected by the *in situ* GH activity assay of fresh mucilage, the decrease in binding intensity by mAb that recognize fucosylated and non-fucosylated xyloglucan carbohydrate epitopes following mild acid hydrolysis of the EIMS, and the increase in binding intensity by mAb that recognize RGI/AG epitopes following mild acid hydrolysis of the EIMS suggested that GH enzymes conferring arabinofuranosidase, fucosidase, galactosidase, mannosidase and xylosidase activities are likely to be important in disassembly of the maize aerial root mucilage polysaccharide.

### Mucilage metagenome analysis using MG-RAST

The microbiota associated with the aerial root mucilage of the Sierra Mixe maize landrace was investigated by analyzing five shotgun metagenome libraries of DNA extracted from mucilage sampled in Mexico. As depicted in the workflow shown in S1 Fig, DNA sequencing reads from all mucilage metagenomes were queried against sequences from the Refseq database using MG-RAST in order to quantify the abundance of ribosomal RNA (rRNA) genes that were present in each mucilage sample [21]. This analysis revealed that the most abundant rRNA genes were those from bacteria (S2 Fig, S1 and S2 Tables). Among the bacterial phyla that were identified within the mucilage metagenomes, the Proteobacteria, Bacteroidetes, Actinobacteria and Firmicutes were found to have the first, second, third and fourth highest relative abundances, respectively. Considering each of the five mucilage metagenomes together, the Proteobacteria classes with the highest relative abundance were Betaproteobacteria and Gammaproteobacteria, which were observed to have similar relative abundance levels. Looking at the Bacteroidetes, the classes Flavobacteria and Sphingobacteria were found to have the highest relative abundance.

Data from the subsystems analysis tool of MG-RAST [13, 27] indicated that the subsystem with the highest relative abundance in each metagenome was determined to be “Carbohydrates” (S3 Fig and S3 Table). This result prompted the application of a subsystem filtering strategy to the metagenome reads that were annotated using MG-RAST, which has been diagramed in S1 Fig. To begin filtering the annotated metagenome reads, a “Carbohydrates” level 1 subsystem filter was applied to the annotated mucilage metagenome sequence reads. Next, the diversity of GH encoding genes within the mucilage metagenomes was achieved by selecting the “Monosaccharides” subsystem. From this point, tertiary subsystem filters were then applied to identify annotated metagenome reads that corresponded to monosaccharides of interest (arabinose, fucose, mannose and xylose) based on the *in-situ* GH activity and mAb cell wall glycan screening assays of aerial root mucilage.

Following the iterative application of tertiary subsystem filters to target protein coding sequences related to the utilization of monosaccharides that were determined to be of interest by the previous mucilage assays, the surviving mucilage metagenome reads were investigated to identify GH encoding genes within the samples. Specifically, reads with predicted GH function that were assigned to each monosaccharide subsystem were evaluated based on the parameters of their sequence alignment to reference sequences from the SEED database. GH sequences of interest for enzyme production and characterization were chosen by selecting those with alignments to mucilage metagenome reads that had the highest relative abundance within the samples, the lowest relative e-value among the candidate metagenome sequences, the highest sequence alignment percent identity, and highest relative bit score. However, because full-length protein coding sequences were required for gene synthesis and the metagenome reads that were annotated by MG-RAST only represented partial gene fragments, the full-length gene sequences from the SEED database of MG-RAST were utilized for gene synthesis instead.

The observed epitope binding events and endogenous glycosyl hydrolase activities of aerial root mucilage from Sierra Mixe maize samples served as guides during the process of mining the previously generated mucilage metagenome sequence libraries to identify GH-encoding genes that were likely to confer functional activities associated with mucilage polysaccharide catabolism. A summary of the reference database matches to the mucilage metagenome queries that were selected from the MG-RAST analysis to acquire complete reference sequences for artificial gene synthesis is presented in Table 1. The five GHs that were selected from this analysis were found to belong to bacterial species in the Proteobacteria or Bacteriodetes phyla. The annotation of these in Uniprot are as follows: α-*N*-arabinofuranosidase (FjArf51), α-L-fucosidase (SlFuc29), β-mannosidase (AfMan2), a xylan β-1,4 xylosidase (SlXyn39) and an oligosaccharide reducing end xylanase (FjXyn8) from *Flavobacterium johnsoniae* UW101, *Spirosoma linguale* DSM 74, *Agrobacterium fabrum* C58, *Spirosoma linguale* DSM 74, and *Flavobaterium johnsoniae* UW101 respectively [22].

These five enzymes proved to be interesting based on the taxonomy of the organisms from which they were found to derive, where previous reports in the literature described taxonomically related bacteria as being associated with grass plants. Specifically, using a 16S ribosomal RNA probing method of different soil samples, Bouffaud et al. reported positive correlations for associations between poaceous plant species and targeted microbial taxa from *Agrobacterium* and *Spirosoma* [28]. In addition, the recent report from Kolton et al. described that Flavobacteria are well suited to survive in environments that are rich in plant-synthesized glycans, which reinforced our decision to pursue the characterization of GHs from the *Flavobacterium johnsoniae* UW101 genome [29]. Results from these studies corroborate the recent comprehensive analysis of genomes derived from plant associated bacteria by Levy et al., which revealed the trend for plant associated bacteria to exhibit diverse enzymatic machinery related to carbohydrate metabolism, thus making them an ideal source for enzyme discovery [30]. While the five predicted gene sequences with putative glycosyl hydrolase function were selected from the aerial root mucilage metagenomes based on their whole genome sequence annotations from the UniProt database, the next step was to validate the predicted function of each enzyme by using a combination of comparative sequence analysis to those with similar annotations and biochemical characterization.

### Phylogenetic analysis of targeted glycosyl hydrolase families

**Fig 4:**
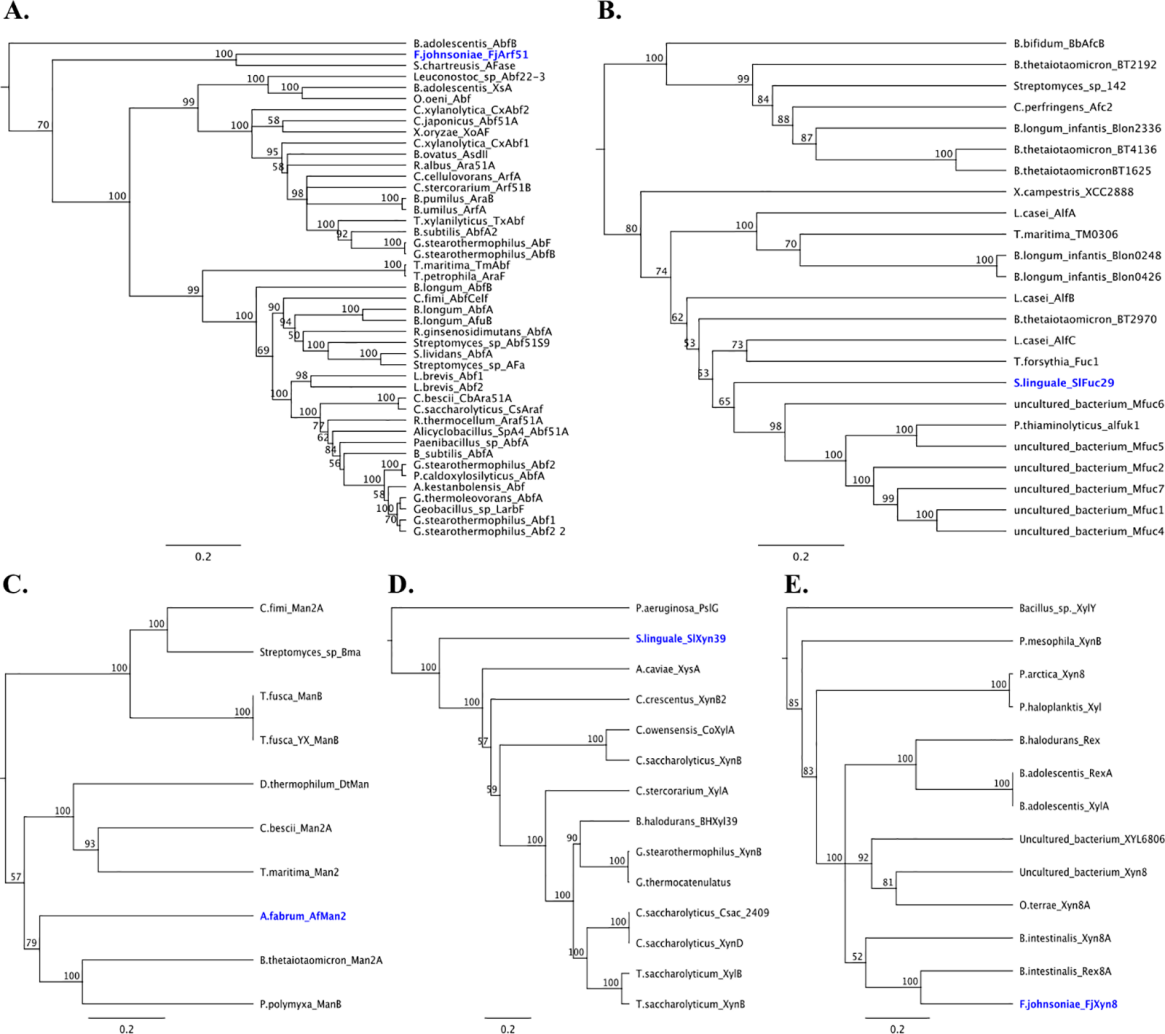
Phylogenetic trees for the glycosyl hydrolase family of each characterized enzyme. The **s**elected full-length protein sequences forming each phylogenetic tree derived from the CAZy database that were listed as “characterized” enzymes with bacteria origin (S4 Table). A) members of GH family 51 with α-N-arabinofuranosidase activity (FjArf51); B) members of GH family 29 with α-L-fucosidase activity (SlFuc29); C) members of GH family 2 with β-mannosidase activity (AfMan2); D) members of GH family 39 with xylan β-1,4-xylosidase activity (SlXyn39); E) members of GH family 8 with oligosaccharide reducing end xylanase activity (FjXyn8).

The CAZy database is a comprehensive repository of information for well characterized bacterial enzymes that includes the classification of GH enzyme sequences under different GH family numbers. Enzymes that are listed under each GH family are then directly linked to the accession pages for the nucleotide and amino acid sequences stored within the NCBI GenBank database. Amino acid sequences for enzymes that corresponded to the same GH family and monosaccharide utilization annotations as those of the enzymes investigated in this study were acquired in FASTA format from the NCBI GenBank database by using the CAZy database to guide the selection of “characterized” (experimental data has been generated resulting in the assignment of an enzyme class number according to IUBMB rules) bacterial enzymes from GH families 2, 8, 29, 39 and 51. The selected “characterized” sequences corresponding to each GH family in this study, were then used to generate multiple sequence alignments and the subsequent phylogenetic trees that are presented in Fig 4. While the CAZy database is a continuously growing database, the number of “characterized” bacterial enzymes present within the database for each GH family was found to be variable during the time of our GH family sequence analysis.

The GH families containing the highest number of “characterized” bacterial enzyme sequences with similar substrate specificities to the targeted enzymes of interest were GH51 and GH29. GH51 had 45 enzyme sequences with proven arabinofuranosidase activity that were incorporated into the tree shown in Fig 4A, including that of the FjArf51 enzyme from *Flavobacterium johnsoniae* UW101. The FjArf51 sequence formed a clade with only one other sequence among the 44 GH51 sequences that were included in the group (an arabinofuranosidase sequence from *Streptomyces chartreusis*) [31]. This result suggested that FjArf51 does not exhibit high sequence similarity to the majority of “characterized” enzymes from GH51, as this pair of sequences (FjArf51 and S. *chartreusis*_AFase) were not found to be deeply nested within either of the major clades on the tree. However, the observed clustering of the FjArf51 sequence with other GH51 sequences from the CAZy database provides one level of confirmation for the sequence annotation-based assignment of putative arabinofuranosidase function.

GH family 29 had the second highest number of bacterial enzyme sequences with biochemically proven fucosidase activity incorporated into its phylogenetic analysis with 24 (SlFuc29 and 23 other sequences) members comprising the tree depicted in Fig 4B. Phylogenetic analysis of these sequences revealed that the sequences clustered as a sister clade to SlFuc29 were assigned “Mfuc” as an enzyme prefix and derived from uncultured bacteria that were previously reported in a soil metagenome study by Lezyk et al. in 2016 [32]. Further investigation into these “Mfuc” sequences revealed that SlFuc29 shared the conserved nucleophile sequence TPEQ with all five “Mfuc” members included in the phylogenetic analysis, while Mfuc1, Mfuc2, Mfuc4 and Mfuc5 all had similar kM values to that of SlFuc29 (0.18) when hydrolyzing 4NP-α-Fuc.

The number of “characterized” bacterial enzyme sequences incorporated into the phylogenetic trees corresponding to GH families 2, 8 and 39 were found to be lower with 10, 14 and 13 sequences included into each group respectively. Fig 4C shows that well-characterized mannosidase sequences from GH2 had the lowest representation among the GH family groups we explored in the CAZy database, where the AfMan2 sequence from *Agrobacterium fabrum* str. C58 formed a clade with two other GH2 mannosidase sequences: one sequence from *Bacteroides thetaiotaomicron* and another from *Paenibacillus polymyxa* [33, 34]. The SlXyn39 enzyme sequence from *Spirosoma linguale* DSM74 was analyzed phylogenetically along with 13 other bacterial enzymes from GH39 with specificities for xylosyl-residues in Fig 4D, which revealed that SlXyn39 was the sister taxon to 12 other sequences with which it formed a monophyletic group. Finally, the sequence for the FjXyn8 enzyme from *Flavobacterium johnsoniae* UW101 was analyzed alongside 12 other well-characterized bacterial enzymes from GH8 with reported activity on xylose-containing sugar molecules. Fig 4E shows that FjXyn8 formed a two-member clade with the Rex8A enzyme from *Bacteroides intestinalis* DSM17393.

### Biochemical characterization of glycosyl hydrolases

The selected GH genes encoding putative GH enzymes from Table 1 were codon optimized for recombinant production in *E*. *coli*, synthetized, and subsequently cloned into the pET-28a (+) vector from Novagen. Protein was then produced by induction using the appropriate final concentration of IPTG (0.1 to 0.4 mM). Cell pellets were enzymatically lysed, and the soluble protein was purified for all five of the recombinant GHs. The amount of soluble protein obtained from 200 mL Terrific Broth cultures differed for each recombinant protein. SlFuc29 had the highest expression level with 25 mg of soluble, purified protein recovered. For the remaining four recombinant proteins, purified recovery totals were as follows: FjArf51 (20 mg), AfMan2 (12.5 mg), SlXyn39 (12.5 mg) and FjXyn8 (10 mg). The purified, soluble protein samples, before dialysis, have been presented in the SDS-PAGE gel image of S4 Fig. Given the gel concentration (12%) and the estimated molecular weight for each of the five purified GH proteins, the total migration distance of each sample was found to be appropriate in relation to the bands of the molecular weight protein standard (S4 Fig, Table 1). Enzyme characterization was carried out for each of the five CAZymes using the corresponding commercial substrates (4NP-substrates). The kinetic constants were calculated at the optimum pH over two temperatures, 30 °C and 50 °C and the data have been summarized in Table 2.

**Table 2:** Determination of kinetic parameters for mucilage derived glycosyl hydrolases. Experiments were run in triplicate under the optimal pH and temperature for each enzyme. Both the enzyme concentration and 4NP substrates concentration varied over ranges of 8–20 μM and 0.2 to 1mM respectively. All reactions were carried out in the corresponding buffer (either citrate-phosphate or phosphate) with a final buffer concentration of 50 mM.

Enzyme	Condition	*K*_M_ (mM)	*k*_cat_ (min^-1^)	*k*_cat/_ *K*_M_ (min^-1^mM^-1^)
α-N-Arabinofuranosidase	4NP-α-Ara 30°C, pH 7	0.22 ± 0.087	6700	31090
α-N-Arabinofuranosidase	4NP-α-Ara 50°C, pH 7	0.46 ± 0.034	10430	22873
α-L-Fucosidase	4NP-α-Fuc 30°C, pH 5	0.58 ± 0.020	3185	5511
α-L-Fucosidase	4NP-α-Fuc 50°C, pH 5	0.18 ± 0.042	9264	52605
β-Mannosidase	4NP-β-Man 30°C, pH 7	0.68 ± 0.219	80980	119018
β-Mannosidase	4NP-β-Man 50°C, pH 7	0.71 ± 0.204	99820	140453
β-Mannosidase	4NP-β-Gal 30°C, pH 7	0.25 ± 0.062	16156	65014
β-Mannosidase	4NP-β-Gal 50°C, pH 7	1.04 ± 0.228	57440	55178
Xylan β-1,4 xylosidase	4NP-β-(Xyl)_3_ 30°C, pH 6	0.08 ± 0.009	130	1615
Xylan β-1,4 xylosidase	4NP-β-(Xyl)_3_ 50°C, pH 6	0.23 ± 0.005	513	2205
Oligosaccharide reducing end xylanase	4NP-β-(Xyl)_3_ 30°C, pH 6	0.790 ± 0.222	309	392
Oligosaccharide reducing end xylanase	4NP-β-(Xyl)_3_ 50°C, pH 6	0.150 ± 0.001	529	3629

The FjArf51 protein showed kinetic parameters that included substrate accommodation with a lower *K*_M_ value when operating at 30 °C, and higher turnover at 30 °C (31090 min^-1^ mM^-1^) when compared to that at 50 °C (22873 min^-1^ mM^-1^). The more relevant enzyme characteristic of FjArf51 was its demonstrated thermostable hydrolytic activity at different temperatures (30 °C,40°C, 50°C and 60 °C) over an incubation period of 40 minutes (S5 Fig). The kinetic parameters for SlFuc29 indicated that the enzyme activity was enhanced at the higher temperature of 50 °C (52605 min^-1^ mM^-1^), where it exhibited a higher turnover rate, and a lower *K*_M_. At first glance, the primary difference between SlFuc29 and the Mfuc enzymes appears to be that SlFuc29 exhibited optimal activity at pH5 and was determined to be thermostable with activity still present as high as 60 °C (S5 Fig), while the Mfuc enzymes reported by Lezyk et al. were shown to display heightened activity at higher pH values that ranged from 7 to 9 but were not found to be thermostable.

The AfMan2 enzyme presented a stable activity at different temperatures with similar turnovers at both 30 °C (119018 min^-1^ mM^-1^) and 50 °C (140453 min^-1^ mM^-1^). Interestingly, this β-mannosidase showed another substrate specificity towards the 4NP-β-Gal, and this secondary activity exhibited a kinetic turnover that was equal to more than half of the kinetic turnover observed with the 4NP-β-Man substrate at both 30 °C (65014 min^-1^ mM^-1^) and 50 °C (55178 min^-1^ mM^-1^). This sequence is classified under GH family 2, which is a family that currently holds 9 “characterized” bacterial members with reported activity on mannose. These sequences were used alongside AfMan2 to construct the phylogenic tree in Fig 4C that depicts AfMan2 within a clade of three taxa on its own branch as a sister taxon to two other sequences included in the analysis. Biochemical assay of AfMan2 revealed that it was active on both the 4NP-β-Man and 4NP-β-Gal substrates (Table 2), which could potentially be explained by the LacZ (COG3250), ebgA (PRK10340), and Glyco_hydro_2 (pfam00703) conserved domains that are present within its protein sequence. This feature makes AfMan2 the first reported β-mannosidase with dual-activity. While the observed activity may potentially be due to the presence of a galactose-binding-like domain, further investigation into the structure and functional promiscuity of this enzyme is required in order to fully elucidate the mechanism by which it is capable of accommodating different hexose isomers as substrates for hydrolysis.

The SlXyn39 demonstrated a higher substrate affinity for 4NP-β-(Xyl)_3_ at 30 °C (*K*_M_ 0.08 and *k*_cat_/ *K*_M_ 1615 min^-1^ mM^-1^), but the higher temperature (50 °C) made the substrate interaction less specific. On the other hand, FjXyn8, with an inverting mechanism, had greater substrate accommodation at higher temperature with the 4NP-β-(Xyl)_3_ substrate at 50 °C (*K*_M_ 0.150 and *k*_cat_/ *K*_M_ 3629 min^-1^ mM^-1^). The use of xylanases from well described GH families such as GH10 and GH11 have been reported for numerous applications, whereas the lack of abundance in well-characterized xylanases from GH families 8 and 39 at both the biochemical and sequence levels may be account for their lack of incorporation into biotechnological processes [10]. Interestingly, the comparative genomic analysis of Kolton et al. described the presence of CAZy genes from GH51 within the genomes of terrestrial Flavobacterium species but does not mention their possession of genes that could encode xylose-acting CAZymes from GH 8 [29]. This development provides further validation of the described strategy to use metagenome sequence analysis in combination with whole genome sequence database mining, where this result has served the purpose of complementing previous reports through the presentation of new information related to GHs from terrestrial species of Flavobacterium.

## Conclusion

This work demonstrates how metagenomic approaches to mine specific environmental samples is a useful strategy for guiding the discovery of gene sequences within large genome sequence databases that may then be utilized for the generation of new enzymatic tools and applications in biotechnology. Furthermore, utilizing the *in-situ* GH activity and non-cellulosic plant cell wall glycan mAb screening assays of aerial root mucilage from Sierra Mixe maize informed the shotgun metagenomic analysis, which expanded the repertoire of knowledge for GH families that derive from plant associated bacteria of terrestrial environments that are rich in complex carbohydrate and provided insight into the enzymatic strategies that are potentially employed by the microbiota of Sierra Mixe maize for the catabolism of mucilage polysaccharide. While a strong indication of taxonomic differences among the key players affiliated with the aerial root mucilage was observed, the findings of the present work also suggest that distinguishable enzymes from within the same microbial ecosystem may work synergistically to hydrolyze this compositionally diverse mucilage polysaccharide. The results from biochemical and phylogenetic analyses of GHs reported in this study serve the scientific community by expanding both the characterization of enzymes derived from microbes associated with plants and the reported biodiversity of the corresponding GH families within the CAZy database. Overall, we highlight the utility of combining metagenomics and synthetic biology to discover and validate enzymatic activities that derive from microbes of geographically isolated ecosystems.

## Supporting information

S1 Fig Diagram of workflow for mucilage metagenome analysis using MG-RAST. Metagenome datasets for the five Sierra Juarez mucilage samples were selected for the MG-RAST analysis. Microbial community analysis was carried out by selecting the Refseq database, which then allowed for the classification of ribosomal sequences and relative abundance estimations of taxa within the samples at different taxonomic levels. The annotation of reads based on function was achieved by selecting the SEED database for subsystems analysis. Level 3 filters that corresponded to monosaccharides related to the aerial root mucilage sugars were applied separately to the sequence reads from all five samples. The filtered reads surviving for each monosaccharide category were then compared based on the relative sample abundance, percent similarity of the alignment to database reference sequences, the identity of positions in the alignments, and the e-value. Reference sequences that aligned to metagenome reads with the lowest e-value, highest sequence similarity, highest identity value and highest relative abundance were then selected for artificial gene synthesis.
(TIFF)

S2 Fig Bacterial rRNA sequences classified by phylum within mucilage metagenomes. The bacterial rRNA sequences within all five aerial root mucilage metagenomes were queried against the Refseq database using MG-RAST version 4.0.3 under the default settings (e-value of 5, 60% identity, length of 15, minimum abundance of 1 and representative hit selected). The metagenome labels on the x-axis correspond to the MG-RAST metagenome reference ID numbers, and the y-axis represents number of annotated sequences.
(TIFF)

S3 Fig Subsystems annotation of mucilage metagenome sequences using MG-RAST. Metagenomic reads were filtered using the MG-RAST analysis tool using the default settings (e-value of 5, 60% identity, length of 15, minimum abundance of 1 and representative hit selected). The metagenome labels on the x-axis correspond to the MG-RAST metagenome reference ID numbers, and the y-axis represents number of annotated sequences.
(TIFF)

S4 Fig Pipeline for protein production and purification. Each lane of the gel image contains the following purified proteins: [L] Precision Plus Protein Kaleidoscope Standard with annotations in kilodaltons (kDa); [1] α-L-Fucosidase (SlFuc29); [2] α-N-Arabinofuranosidase (FjArf51); [3] β-Mannosidase (AfMan2); [4] Oligosaccharide reducing end xylanase (FjXyn8); [5] Xylan β-1,4 xylosidase (SlXyn39).
(TIFF)

S5 Fig Assays to identify optimal conditions for enzyme activity. Enzyme activity assays were carried out in order to identify optimal temperature and pH conditions. Three enzymes were assayed for temperature optima: A) α-N-Arabinofuranosidase (FjArf51), B) α-L-Fucosidase (SlFuc29), C) β-Mannosidase (AfMan2). The two xylan acting enzymes were assayed for pH optima: D) Xylan β-1,4 xylosidase (SlXyn39) and E) Oligosaccharide reducing end xylanase (FjXyn8).
(TIFF)

S1 Table Number of mucilage metagenomic sequence matches to the Refseq database. The MG-RAST analysis tool (version 4.0.3) was used to assess the relative abundance of phyla within the mucilage metagenomes. Each of the five mucilage metagenome samples are indicated by their MG-RAST reference ID number. The values represent the number of query sequences from each metagenome that matched sequences in the Refseq database.
(DOCX)

S2 Table Refseq hits ranked by class for bacterial phyla with high relative abundance. The metagenome query sequence matches to the Refseq database produced using the MG-RAST analysis tool (version 4.0.3) were classified by the associated record’s microbial class. Each of the five mucilage metagenome samples are indicated by their MG-RAST reference ID number. The values represent the number of query sequences from each metagenome that matched sequences in the Refseq database.
(DOCX)

S3 Table Subsystems annotation of aerial root mucilage metagenomes using MG-RAST. Metagenome sequence queries were annotated using the MG-RAST subsystems database and the data summarized was generated using the analysis tool feature (version 4.0.3). Each of the five mucilage metagenome samples are indicated by their MG-RAST reference ID number.
(DOCX)

S4 Table DNA sequences of the codon optimized synthetic genes. Nucleotide sequences were acquired from NCBI Genbank and were codon optimized (red colored nucleotides) for artificial gene synthesis and cloning into the pET-28a(+) vector (Novagen) by Genscript Inc. (Piscataway, New Jersey).
(DOCX)

S5 Table Protein sequences used for phylogenetic analysis of glycosyl hydrolase families. The following sequences were downloaded from NCBI GenBank after browsing the CAZy database and were incorporated into the phylogenetic analysis used to generate the trees shown in Fig 4.
(DOCX)
