# Supplementary figures and images for: Characterization of novel glycosyl hydrolases discovered by cell wall glycan directed monoclonal antibody screening and metagenome analysis of maize aerial root mucilage
Source: PLoS One. 2018 Sep 26;13(9):e0204525. doi: 10.1371/journal.pone.0204525 (PMC6157868; doi:10.1371/journal.pone.0204525)

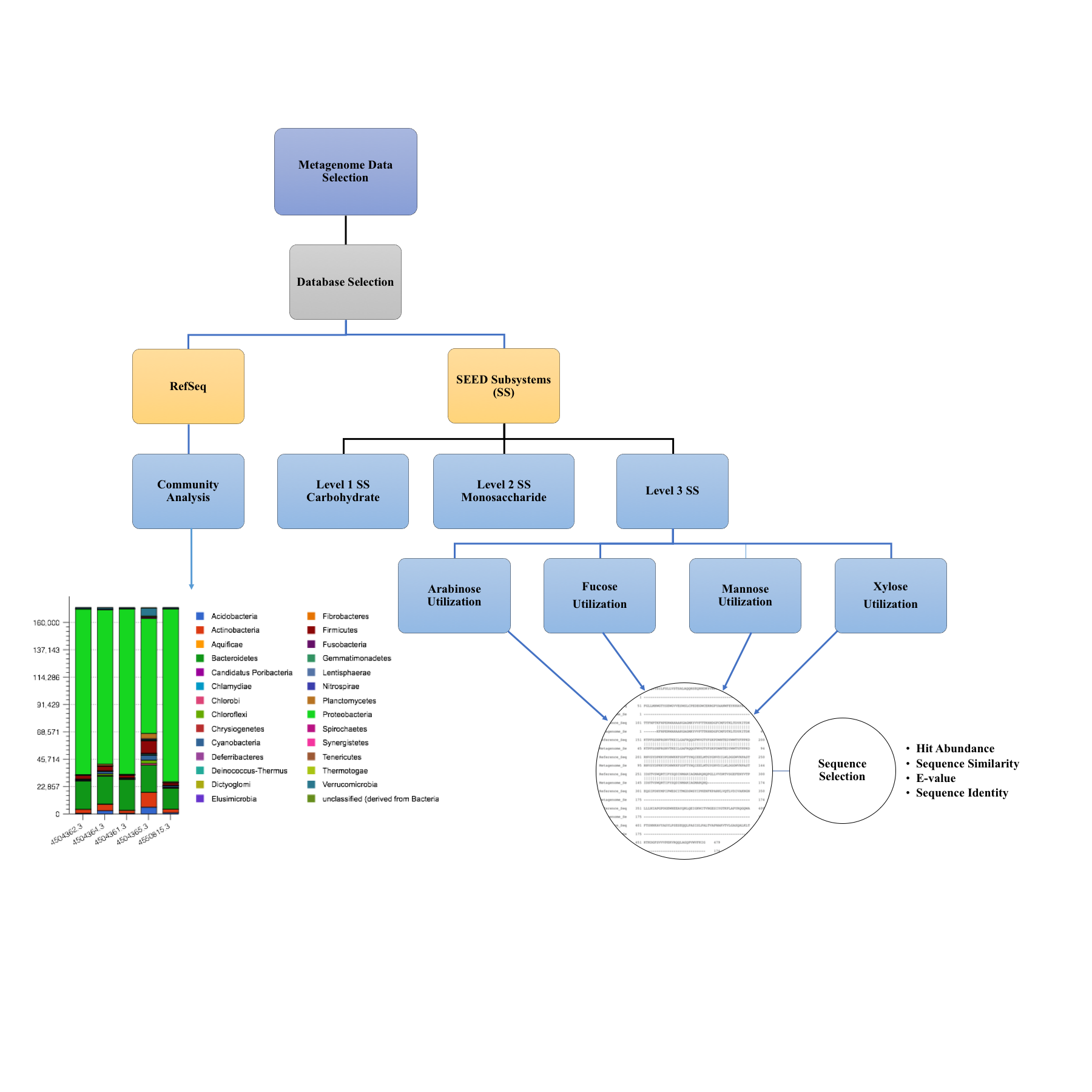

Supplement: S1 Fig — Metagenome datasets for the five Sierra Juarez mucilage samples were selected for the MG-RAST analysis. Microbial community analysis was carried out by selecting the Refseq database, which then allowed for the classification of ribosomal sequences and relative abundance estimations of taxa within the samples at different taxonomic levels. The annotation of reads based on function was achieved by selecting the SEED database for subsystems analysis. Level 3 filters that corresponded to monosaccharides related to the aerial root mucilage sugars were applied separately to the sequence reads from all five samples. The filtered reads surviving for each monosaccharide category were then compared based on the relative sample abundance, percent similarity of the alignment to database reference sequences, the identity of positions in the alignments, and the e-value. Reference sequences that aligned to metagenome reads with the lowest e-value, highest sequence similarity, highest identity value and highest relative abundance were then selected for artificial gene synthesis. (TIFF) [file pone.0204525.s001.tiff]

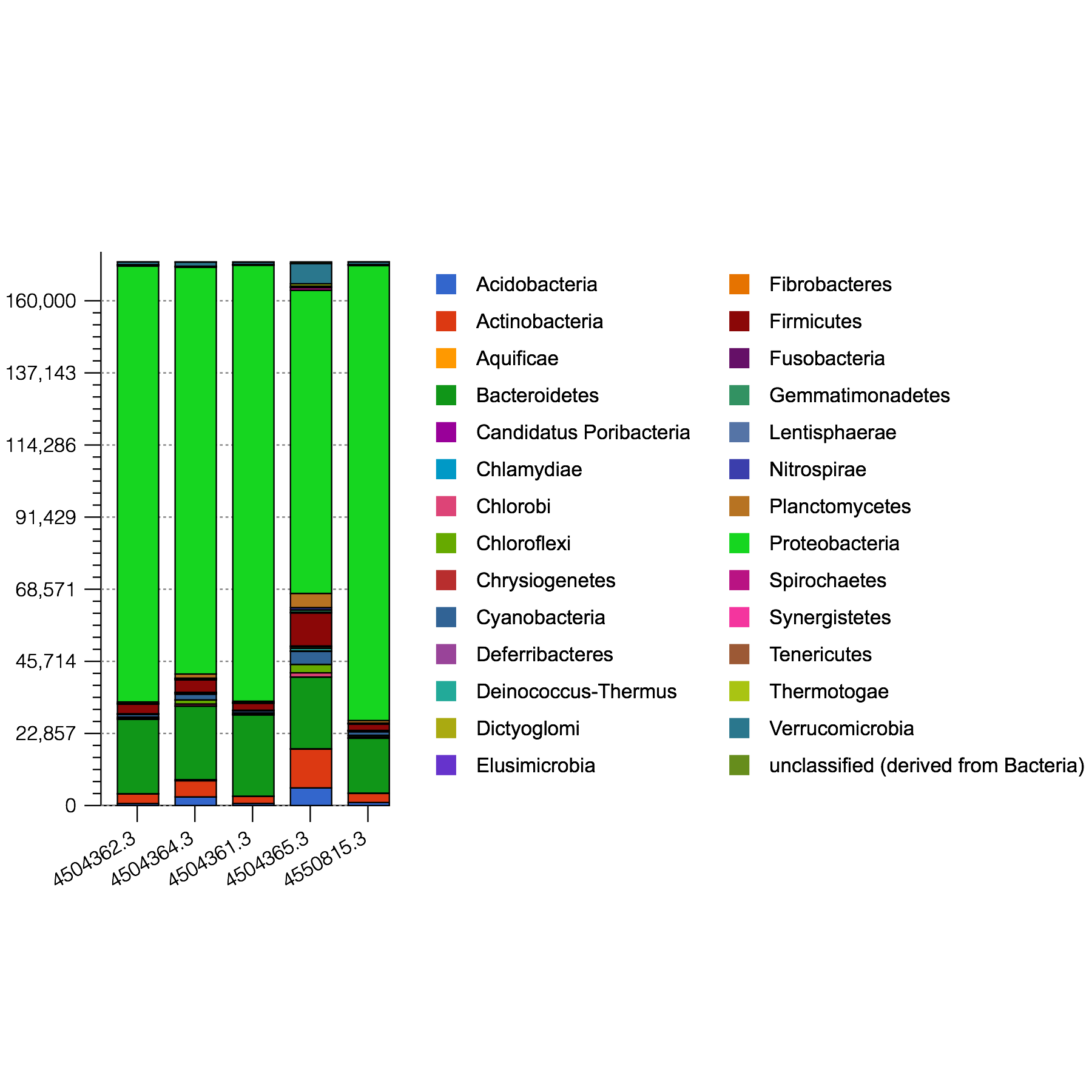

Supplement: S2 Fig — The bacterial rRNA sequences within all five aerial root mucilage metagenomes were queried against the Refseq database using MG-RAST version 4.0.3 under the default settings (e-value of 5, 60% identity, length of 15, minimum abundance of 1 and representative hit selected). The metagenome labels on the x-axis correspond to the MG-RAST metagenome reference ID numbers, and the y-axis represents number of annotated sequences. (TIFF) [file pone.0204525.s002.tiff]

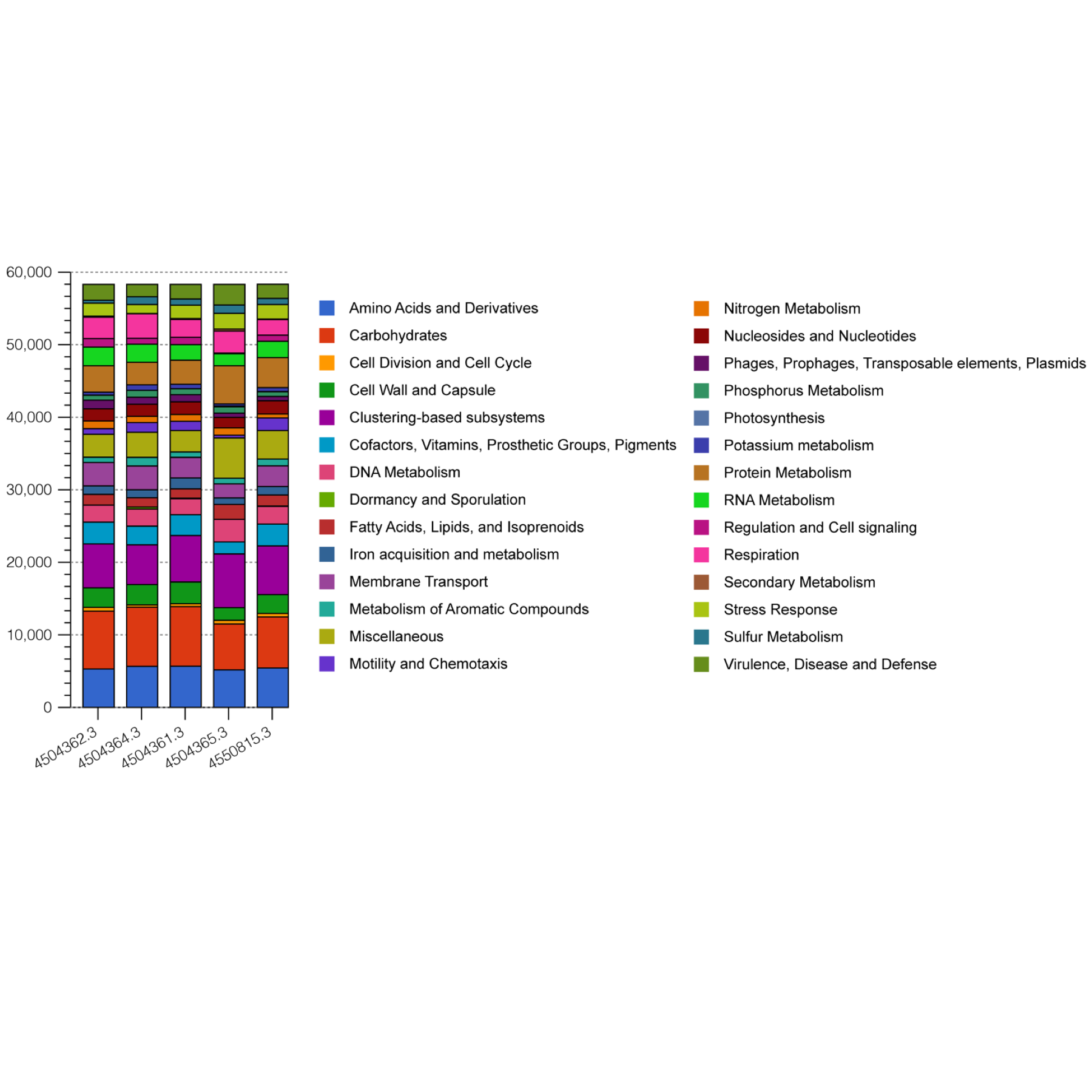

Supplement: S3 Fig — Metagenomic reads were filtered using the MG-RAST analysis tool using the default settings (e-value of 5, 60% identity, length of 15, minimum abundance of 1 and representative hit selected). The metagenome labels on the x-axis correspond to the MG-RAST metagenome reference ID numbers, and the y-axis represents number of annotated sequences. (TIFF) [file pone.0204525.s003.tiff]

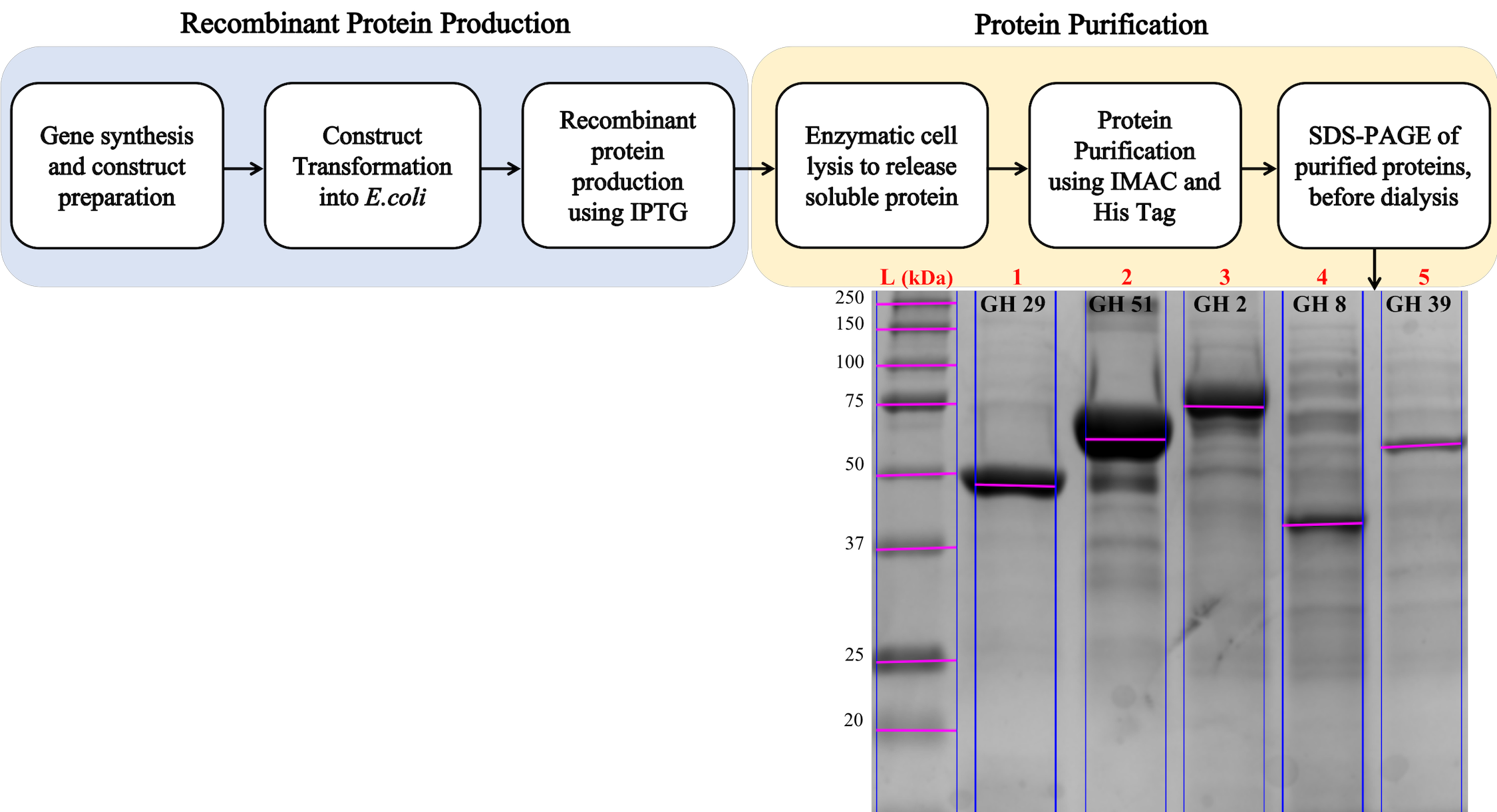

Supplement: S4 Fig — Each lane of the gel image contains the following purified proteins: [L] Precision Plus Protein Kaleidoscope Standard with annotations in kilodaltons (kDa); [1] α-L-Fucosidase (SlFuc29); [2] α-N-Arabinofuranosidase (FjArf51); [3] β-Mannosidase (AfMan2); [4] Oligosaccharide reducing end xylanase (FjXyn8); [5] Xylan β-1,4 xylosidase (SlXyn39). (TIFF) [file pone.0204525.s004.tiff]

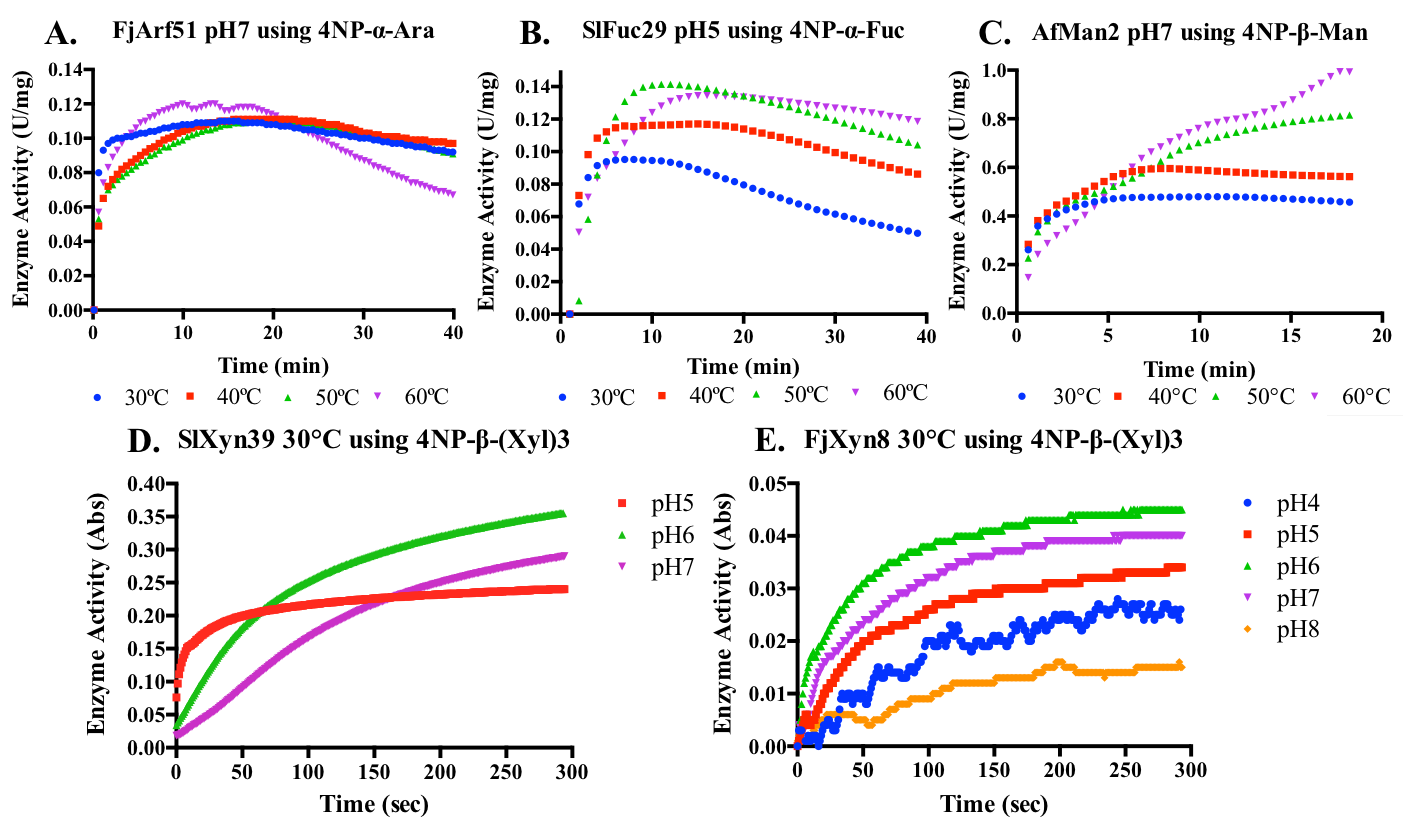

Supplement: S5 Fig — Enzyme activity assays were carried out in order to identify optimal temperature and pH conditions. Three enzymes were assayed for temperature optima: A) α-N-Arabinofuranosidase (FjArf51), B) α-L-Fucosidase (SlFuc29), C) β-Mannosidase (AfMan2). The two xylan acting enzymes were assayed for pH optima: D) Xylan β-1,4 xylosidase (SlXyn39) and E) Oligosaccharide reducing end xylanase (FjXyn8). (TIFF) [file pone.0204525.s005.tiff]
