# Supplementary material for: Characterization of novel glycosyl hydrolases discovered by cell wall glycan directed monoclonal antibody screening and metagenome analysis of maize aerial root mucilage
Source: PLoS One. 2018 Sep 26;13(9):e0204525. doi: 10.1371/journal.pone.0204525 (PMC6157868; doi:10.1371/journal.pone.0204525)
Supplement: S1 Table — The MG-RAST analysis tool (version 4.0.3) was used to assess the relative abundance of phyla within the mucilage metagenomes. Each of the five mucilage metagenome samples are indicated by their MG-RAST reference ID number. The values represent the number of query sequences from each metagenome that matched sequences in the Refseq database. (DOCX) [file pone.0204525.s006.docx]

| Domain | Phylum | mgm 4504362.3 | mgm 4504364.3 | mgm 4504361.3 | mgm 4504365.3 | mgm 4550815.3 |
| --- | --- | --- | --- | --- | --- | --- |
| Bacteria | Acidobacteria | 57 | 76 | 107 | 46 | 840 |
| Bacteria | Acidobacteria | 270 | 142 | 359 | 101 | 2520 |
| Eukaryota | Apicomplexa | 2 | 0 | 12 | 1 | 5 |
| Bacteria | Aquificae | 4 | 10 | 6 | 1 | 86 |
| Eukaryota | Arthropoda | 5 | 4 | 25 | 12 | 50 |
| Eukaryota | Ascomycota | 97 | 3 | 686 | 4 | 112 |
| Eukaryota | Bacillariophyta | 0 | 2 | 4 | 0 | 10 |
| Bacteria | Bacteriodetes | 2082 | 647 | 4104 | 187 | 15216 |
| Eukaryota | Basidiomycota | 12 | 1 | 37 | 0 | 18 |
| Bacteria | Candidatus Poribacteria | 0 | 0 | 2 | 0 | 12 |
| Bacteria | Chlamydiae | 6 | 2 | 7 | 0 | 63 |
| Bacteria | Chlorobi | 27 | 17 | 42 | 11 | 302 |
| Bacteria | Chloroflexi | 30 | 35 | 43 | 22 | 423 |
| Eukaryota | Chlorophyta | 4 | 2 | 14 | 0 | 42 |
| Eukaryota | Chordata | 18 | 8 | 114 | 0 | 233 |
| Bacteria | Chrysiogenetes | 0 | 1 | 4 | 0 | 33 |
| Eukaryota | Cnidaria | 11 | 7 | 48 | 2 | 1165 |
| Archaea | Crenarchaeota | 0 | 2 | 9 | 1 | 13 |
| Bacteria | Cyanobacteria | 75 | 50 | 100 | 34 | 959 |
| Bacteria | Deferribacteres | 3 | 6 | 6 | 1 | 53 |
| Bacteria | Deinococcus-Thermus | 14 | 8 | 26 | 7 | 307 |
| Bacteria | Dictyoglomi | 0 | 1 | 0 | 4 | 10 |
| Eukaryota | Echinodermata | 0 | 0 | 0 | 0 | 2 |
| Bacteria | Elusimicrobia | 1 | 0 | 3 | 2 | 5 |
| Archaea | Euryarchaeota | 7 | 9 | 17 | 10 | 191 |
| Bacteria | Fibrobacteres | 0 | 2 | 0 | 0 | 10 |
| Bacteria | Firmicutes | 268 | 109 | 351 | 86 | 1756 |
| Bacteria | Fusobacteria | 9 | 4 | 15 | 2 | 81 |
| Bacteria | Gemmatimonadetes | 7 | 7 | 7 | 6 | 100 |
| Eukaryota | Hemichordata | 0 | 1 | 1 | 0 | 0 |
| Archaea | Korarchaeota | 0 | 0 | 0 | 0 | 1 |
| Bacteria | Lentisphaerae | 2 | 2 | 3 | 0 | 47 |
| Eukaryota | Microsporidia | 8 | 1 | 0 | 0 | 29 |
| Eukaryota | Nematoda | 2 | 0 | 7 | 0 | 13 |
| Bacteria | Nitrospirae | 2 | 3 | 7 | 6 | 65 |
| Eukaryota | Placozoa | 0 | 0 | 0 | 0 | 3 |
| Bacteria | Planctomycetes | 42 | 36 | 73 | 37 | 693 |
| Eukaryota | Platyhelminthes | 0 | 0 | 0 | 0 | 1 |
| Bacteria | Proteobacteria | 12191 | 3584 | 21987 | 790 | 126161 |
| Bacteria | Spirochaetes | 32 | 7 | 24 | 7 | 222 |
| Eukaryota | Streptophyta | 188 | 21 | 294 | 0 | 736 |
| Bacteria | Synergistetes | 2 | 0 | 2 | 1 | 29 |
| Bacteria | Tenericutes | 2 | 0 | 3 | 3 | 15 |
| Archaea | Thaumarchaeota | 2 | 0 | 3 | 6 | 5 |
| Bacteria | Thermotogae | 2 | 2 | 9 | 6 | 51 |
| Bacteria | unclassified (derived from Bacteria) | 2 | 2 | 11 | 4 | 40 |
| Eukaryota | unclassified (derived from Eukaryota) | 12 | 5 | 11 | 1 | 70 |
| Other sequences | unclassified (derived from other sequences) | 0 | 0 | 0 | 0 | 4 |
| Viruses | unclassified (derived from Viruses) | 39 | 16 | 34 | 5 | 114 |
| Bacteria | Verrucomicrobia | 75 | 36 | 120 | 53 | 694 |
