# Supplementary material for: Characterization of novel glycosyl hydrolases discovered by cell wall glycan directed monoclonal antibody screening and metagenome analysis of maize aerial root mucilage
Source: PLoS One. 2018 Sep 26;13(9):e0204525. doi: 10.1371/journal.pone.0204525 (PMC6157868; doi:10.1371/journal.pone.0204525)
Supplement: S2 Table — The metagenome query sequence matches to the Refseq database produced using the MG-RAST analysis tool (version 4.0.3) were classified by the associated record’s microbial class. Each of the five mucilage metagenome samples are indicated by their MG-RAST reference ID number. The values represent the number of query sequences from each metagenome that matched sequences in the Refseq database. (DOCX) [file pone.0204525.s007.docx]

| Domain | Phylum | Class | mgm  4504362.3 | mgm  4504364.3 | mgm  4504361.3 | mgm  4504365.3 | mgm  4550815.3 |
| --- | --- | --- | --- | --- | --- | --- | --- |
| Bacteria | Actinobacteria | Actinobacteria (class) | 270 | 142 | 359 | 101 | 2520 |
| Bacteria | Bacteroidetes | Bacteroidia | 243 | 57 | 359 | 26 | 1133 |
| Bacteria | Bacteroidetes | Cytophagia | 297 | 114 | 468 | 33 | 4241 |
| Bacteria | Bacteroidetes | Flavobacteria | 1085 | 287 | 2539 | 92 | 4275 |
| Bacteria | Bacteroidetes | Sphingobacteria | 444 | 185 | 702 | 35 | 5504 |
| Bacteria | Bacteroidetes | unclassified (derived from Bacteroidetes) | 13 | 4 | 36 | 1 | 63 |
| Bacteria | Firmicutes | Bacilli | 119 | 52 | 217 | 49 | 843 |
| Bacteria | Firmicutes | Clostridia | 140 | 53 | 124 | 35 | 859 |
| Bacteria | Firmicutes | Erysipelotrichi | 2 | 1 | 5 | 1 | 12 |
| Bacteria | Firmicutes | Negativicutes | 7 | 3 | 5 | 1 | 42 |
| Bacteria | Proteobacteria | Alphaproteobacteria | 1820 | 891 | 3581 | 228 | 27825 |
| Bacteria | Proteobacteria | Betaproteobacteria | 4334 | 1087 | 7530 | 227 | 65221 |
| Bacteria | Proteobacteria | Deltaproteobacteria | 164 | 132 | 281 | 78 | 8414 |
| Bacteria | Proteobacteria | Epsilonproteobacteria | 14 | 11 | 51 | 3 | 157 |
| Bacteria | Proteobacteria | Gammaproteobacteria | 5853 | 1461 | 10532 | 253 | 24404 |
| Bacteria | Proteobacteria | Zetaproteobacteria | 4 | 1 | 2 | 0 | 24 |
| Bacteria | Proteobacteria | unclassified (derived from Proteobacteria) | 2 | 1 | 10 | 1 | 116 |
