# Supplementary material for: Characterization of novel glycosyl hydrolases discovered by cell wall glycan directed monoclonal antibody screening and metagenome analysis of maize aerial root mucilage
Source: PLoS One. 2018 Sep 26;13(9):e0204525. doi: 10.1371/journal.pone.0204525 (PMC6157868; doi:10.1371/journal.pone.0204525)
Supplement: S3 Table — Metagenome sequence queries were annotated using the MG-RAST subsystems database and the data summarized was generated using the analysis tool feature (version 4.0.3). Each of the five mucilage metagenome samples are indicated by their MG-RAST reference ID number. (DOCX) [file pone.0204525.s008.docx]

| Subsystems-Level 1 | mgm4504362.3 | mgm4504364.3 | mgm4504361.3 | mgm4504365.3 | mgm4550815.3 |
| --- | --- | --- | --- | --- | --- |
| Amino Acids and Derivatives | 565 | 186 | 1128 | 53 | 5432 |
| Carbohydrates | 854 | 268 | 1627 | 65 | 7048 |
| Cell Division and Cell Cycle | 56 | 10 | 75 | 5 | 476 |
| Cell Wall and Capsule | 287 | 93 | 598 | 18 | 2576 |
| Clustering-based subsystems | 646 | 180 | 1268 | 76 | 6743 |
| Cofactors, Vitamins, Prosthetic Groups, Pigments | 322 | 84 | 568 | 17 | 2995 |
| DNA Metabolism | 250 | 78 | 439 | 32 | 2432 |
| Dormancy and Sporulation | 2 | 9 | 9 | 0 | 61 |
| Fatty Acids, Lipids, and Isoprenoids | 156 | 42 | 257 | 21 | 1527 |
| Iron acquisition and metabolism | 125 | 36 | 298 | 9 | 1169 |
| Membrane Transport | 346 | 108 | 566 | 20 | 2833 |
| Metabolism of Aromatic Compounds | 78 | 39 | 143 | 8 | 951 |
| Miscellaneous | 338 | 114 | 591 | 57 | 3933 |
| Motility and Chemotaxis | 82 | 44 | 250 | 4 | 1722 |
| Nitrogen Metabolism | 114 | 28 | 185 | 10 | 542 |
| Nucleosides and Nucleotides | 178 | 55 | 347 | 15 | 1822 |
| Phages, Prophages, Transposable elements, Plasmids | 127 | 32 | 196 | 6 | 595 |
| Phosphorus Metabolism | 72 | 31 | 161 | 9 | 656 |
| Photosynthesis | 3 | 0 | 2 | 1 | 24 |
| Potassium metabolism | 44 | 25 | 120 | 3 | 540 |
| Protein Metabolism | 390 | 102 | 658 | 54 | 4127 |
| RNA Metabolism | 275 | 82 | 423 | 17 | 2255 |
| Regulation and Cell signaling | 124 | 27 | 204 | 1 | 858 |
| Respiration | 315 | 111 | 484 | 31 | 2127 |
| Secondary Metabolism | 16 | 1 | 26 | 3 | 96 |
| Stress Response | 191 | 41 | 368 | 22 | 1981 |
| Sulfur Metabolism | 45 | 35 | 166 | 12 | 852 |
| Virulence, Disease and Defense | 232 | 56 | 400 | 29 | 1944 |
