# Supplementary material for: Characterization of novel glycosyl hydrolases discovered by cell wall glycan directed monoclonal antibody screening and metagenome analysis of maize aerial root mucilage
Source: PLoS One. 2018 Sep 26;13(9):e0204525. doi: 10.1371/journal.pone.0204525 (PMC6157868; doi:10.1371/journal.pone.0204525)
Supplement: S4 Table — Nucleotide sequences were acquired from NCBI Genbank and were codon optimized (red colored nucleotides) for artificial gene synthesis and cloning into the pET-28a(+) vector (Novagen) by Genscript Inc. (Piscataway, New Jersey). (DOCX) [file pone.0204525.s009.docx]

| Glycosyl Hydrolase | Enzyme Name | Codon optimized sequence |
| --- | --- | --- |
| α-N-Arabinofuranosidase | FjArf51 | 9 AAAAGTATCTTCGGCTGCCTGCTGCTGAGTTCACTGTACACCAATGCACAAAAAGCTAAT  69 CTGGAAGTTGACGCAAGTAAAACGATCACCAAAATCCAGCCGACGATGTTTGGCCTGTTT  129 TTCGAAGATATTAACTTCGCGGCCGACGGCGGTCTGTACGCAGAAATGATCAAAAACCGT  129 TTTGAGGATATCAATTTTGCTGCAGATGGCGGATTATATGCCGAAATGATTAAAAACCGC  189 AGTTTCGAATTCGATAAACCGATGATGGGCTGGGAACAGCCGAACACCAAACGCAGCTCT  249 CTGAATAAAGAAAGTGGTTCCGCGCTGCCGATTAACCTGAGTAAAGAGAAAAACAACTCC  309 AACTTCTGCCGTGTTGAAATCAACAATGATAAAGGCTATACCCTGATTAACGAAGGTTTC  369 CGCGGCATGGGTGTGAAAAAAGACGCCAAATACAATCTGAGCCTGAAAGTTGCCAACCAT  429 AACGGCGCAATTAAGAAAATTATCTTCCAGCTGATCAACAAAGATCAAAAAATCATCGGT  489 GAAACCTCAATCGTGCCGAAATCGGAACAGTGGACCAATTATACGTCGCAATTTACCGCA  549 GTTGAAACGGAAGCGAAAGCCAAACTGAAAATCACCTTTGAAGGCAACGGTACGATTGAT  609 CTGGACATGGTGTCTCTGTTCCCGGAAGATACCTGGAAAAACCGTAAAAATGGCCTGCGC  669 AAAGATCTGGTTCAGCTGCTGTATGACGTCAAACCGGGCTTTCTGCGTTTCCCGGGCGGT  729 TGTATTGTCGAAGGTCGTACCCTGAGCGATCGCTACCAGTGGAAAAAATCTGTGGGTGAC  789 GTCGAAGAACGTAAAACCATGATGAACCGCTGGAATGTGGAATTTAATCATAAACAGACG  849 CCGGATTATTTTCAAAGCTTCGGCCTGGGCTTTTTCGAATACTTCCAGCTGTCTGAAGAT  909 ATCGGCGCGGAACCGCTGCCGATTCTGAGTTGCGGCATGGCCTGTCAGTATAACACCGGT  969 GAACTGGCACCGATGGATGAACTGGACCCGTACATCCAAGATGCTCTGGACCTGATTGAA  1029 TTTGCGAATAGCGATGTTTCTACGAAATGGGGCAAACTGCGTTCCGACATGGGTCACCCG  1089 AAACCGTTCAACCTGAAATATATTGGCGTTGGTAATGAACAGTGGGGCAAAGATTATATC  1149 GACCGCTACAAAGTCTTTGAAAAAGCGATCAAAGCCAAATACCCGAAAATCATCATCGTG  1209 AGCGGCACCGGTCCGTCTCCGAAAGGTGAACATTTCGATTACGCTATGACGGAACTGAAA  1269 AAACTGAACGCGGAACTGATCGACGAACACTATTACGAATCTCCGAAATGGTTTCGTGAA  1329 AACGCGGGTCGCTATGATAATTACGACCGTAAAGGCCCGAAAATTTTCGCCGGTGAATAC  1389 GCAGCTCAGTCAGTTTCGGGCGCAAATCCGAACAATCGTAACAATTGGGAATGCGCTTTT  1449 TCCGAAGCGGCCTTCATGACCGGTCTGGAACGCAACGCCGAAGTGGTTAATATGACGTCA  1509 TATGCACCGCTGATGGCTCACGAAGATGCGTGGCAGTGGACCCCGGACATGATTTGGTTT  1569 AACAATCTGCAAAGTTACGGCTCCGCAAACTATTACGTGCAGCAACTGTTCAGTACCAAT  1569 AATAATCTGCAGTCATATGGTTCTGCAAATTATTATGTGCAGCAATTGTTCTCAACCAAT  1629 AAGGGTACGGATCTGCTGTCCATCACGCAGGACGGCAAAGCTCTGATTGGTCAAAACAAT  1629 AAAGGAACAGATTTGTTAAGCATTACACAGGACGGCAAAGCTTTAATTGGTCAGAACAAT  1689 CTGTATGCATCAGCTGTGAAAGATGTTAACTCGAAAGAAATCATTGTCAAACTGGTGAAC  1749 ACCGCAGCTACGAATCAGGAAGTTAGCATCGATCTGAAAGGCGCGAAACTGGGCTCAAAA  1809 GGTTCGGTCATTAGCCTGGCCTCTGGTAACCTGCAGGATGAAAATACCTTTGTCGAACCG  1869 CGCAAAATCAGTCCGAAACAATCCGAATATAAAGTGACCAAAGGTTCACAGCAACTGACG  1929 CTGCCGGCCTACTCGGTCACCGTGCTGAAACTGAAAACGATT |
| α-L-Fucosidase | SlFuc29 | 9 ATTCTGTTCTCACTGCTGGTCTCGACGAGCGCACTGGCACAACAACATAGCGAACAAAAT  69 CACGATAAATACGTCTGGCCGAAAGATGAACTGGTGAAGAAAAAACTGGCTAACTGGCAG  129 GATATTAAATTTGGCCTGCTGATGCATTGGGGTACCTATTCTGAATGGGGCGTGGTTGAA  189 AGTTGGTCCCTGTGCCCGGAAGACGAAGGTTGGTGTGAACGTCGCGGTCCGTACGCAGCA  249 AACTGGTTCGAATACAAAAAAGCGTACGAAAATCTGCAGACCACGTTTAACCCGACCAAA  309 TTCAATCCGGAACGTTGGGCCAATGCAGCTAAAGGCGCCGGTATGAAATATGTCGTGTTT  369 ACCACGAAACATCACGATGGTTTTTGCATGTTCGATACCAAACTGACGGACTACAAAATT  429 ACCGATAAGAAAACCCCGTTCAGCTCTAACCCGCGTAGCAATGTCACCAAAGAAATCCTG  489 GGCGCCTTTCGCCAGCAAGGCTTCATGGTGGGCACGTATTTTAGCAAACCGGACTGGCAT  549 ACCGAAGATTATTGGTGGACGTACTTCCCGCCGAAAGACCGCAACGTTTCTTATGATCCG  609 AAAAAATACCCGGACCACTGGAAAAAATTCAGTGATTTCACCTACAACCAGATTGAAGAA  669 CTGATGACGGGCTACGGTAATGTCGACATCCTGTGGCTGGATGGCGGTTGGGTGCGTCCG  729 GCATCCACCATTGATTCAACGGTTTCGTGGCAGCGCACCATCCCGTATAGTCAAGACATT  789 AACATGGCGCGTATCGCCGGTATGGCACGCCAGCATCAACCGGGCCTGCTGGTTGTCGAT  789 AACATGGCACGCATTGCCGGTATGGCGCGCCAGCATCAGCCGGGCCTTCTGGTTGTTGAC  849 CGTACCGTGAGTGGTGAATTTGAAAATTATGTTACGCCGGAACAGTCCATTCCGGATCAC  849 CGGACGGTATCGGGCGAATTCGAGAATTACGTCACCCCGGAACAGTCTATCCCCGACCAT  909 TACATGCCGATTCCGTGGGAATCATGTATCACGATGGGCGACTCATGGTCGTATATCCCG  909 TACATGCCCATTCCCTGGGAAAGCTGCATCACTATGGGCGATAGTTGGTCGTACATCCCT  969 AAAGAAAACTTCAAACCGGCCCGCAAACTGGTGCAGACCCTGGTTGATATTGTCGCTAAA  1029 AACGGTAATCTGCTGCTGAATATCGCACCGGGTCCGGACGGTGAATGGCACGAAGAAGCA  1089 TACCAGCGTCTGCAAGAAATTGGTAAATGGATCACCGTTAACGGCGAAAGCATTTATGGT  1149 ACGAAACCGCTGGCACCGTACCGTCAGGGTCAATGGGCATTTACCAGCAACAATAAAGCT  1209 GTTTATGCGTCTTACCTGCCGAGCGAATCTGAACAGCAACTGCCGGCTAGTATCTCCCTG  1269 CCGGCACTGACCGTCGCACCGAATGCAAAAGTGACGGTTCTGGGTGCTTCGCAGGCGCTG  1329 AAACTGACCAAAACGAAAGATGGCTTTAGCGTGGTTGTCCCGGAAAAAGTGCGTCAGCAA  1389 CTGGCCGGTCAACCGGTCTGGGTGTTCAAAATTGGC |
| β-Mannosidase | AfMan2 | 9 ACCGTCATCGACCTGGCTGGCCTGTGGCATCTGGCGAGTGTTGAAGGCGACCACGCTACC  69 GAAATCAGTATTCCGGGCGACATCCATTCTGCACTGAAAAACGCGGCCATTATCCCGGAC  129 CCGTATCATGGTGCAAATGAAAAAGCTGTGCAGTGGGTTGCGCAGCAAGATTGGATTATC  189 GAACGTACCTTTATTCTGGATGACGCAGAAGCTAGTTGGTATCTGGATATTGACTACCTG 249 GATACGGTTGCTATCGTTTTTGTCAACGATGTGCCGGTTCTGTCGGCGGACAATTGCTTC  309 CGTCGCTATCGTCCGGATATTAGCCGTGCGGTGCGCCCGGGTGAAAACACCATTCGCATC  369 CATTTTCACTCTAATATCACGGCAGGTGCAGAACGTCAGGCACGTCAACCGTTCTATATT  429 CCGTACCATCCGGGTAACAGTCCGATCGCAAACGGCAATATGCTGCGTAAACCGCAGTGT  489 CACTTTGGCTGGGACTGGAATATTGCAATCGCTCCGCTGGGTCTGTATGGCAAAATTCTG  549 CTGAAACGTCTGGATACCGCGCGCATCGAACATGTGGTTAGCTCTCAGCATCACGTTGAA  609 GGCGGTGTCGAACTGCACGTCGCAGTGACCCTGTTCGCAGAAGGTCCGGCCTCACTGCCG  669 GTTTATCTGTCGCTGGGCGATGAACGTCTGCGTCTGGAATGCGGTGTGGGTGCTGGTGAA  729 ACCGTCGTGCGCCATGTCTTTTTCGTGGAAAACCCGGATCTGTGGTGGCCGGCAGGTTCT  789 GGCGAACAGACCCTGTACAAACTGACGGTTGAACTGCCGGATGAAACCGTCACGCGTCAA  849 ATTGGTTTTCGCACCATCGAACTGCTGACGGATAAAGACGAAGCCGGCAGTCGCTTTGCA  909 TTCCGTATTAACGGTCGCGAAATCTTCTGCCGTGGCGCGAATTGGATTCCGGCGGACGCC  969 CTGTATTCACTGACCTCGCGCGAAAAAACGGAAGATCTGCTGTGTAGCGCAGTTGAAGCT  1029AACATGAATATGATCCGTGTCTGGGGCGGTGGCTTTTATGAAGAAGACTGGTTCTACGAC  1089CTGTGCGATCGCCTGGGTCTGCTGGTGTGGCAGGATTTTATGTTCGCGTGCAACCTGTAT  1149CCGTGTAGCGAAGACTTTCTGGATAATGTGGAACATGAAGTTGACTACCAAGTCAAACGT  1209CTGAGTTCCCACCCGTCGATCGCCCTGTGGTGTGGTGATAACGAACTGGTTGGCGCACTG  1269ACCTGGTTCGATGAAAGCCGCAACAATCGTGACCGCTATCTGGTGGCCTACGATCGTCTG  1329AATCGCACCATTGAAAAAGCACTGAAAAAAGCTACGCCGGAAGCACTGTGGTGGCCGTCA  1389TCGCCGGCAAGCGGTTATCTGGACTACGGTGATGCATGGCATGCAGACGGTAGCGGCGAT  1449ATGCATTATTGGTCTGTCTGGCACGAAAACAAAAGTTTTGATAATTACCACCAGGTGAAA  1509CCGCGTTTCTGCTCCGAATTTGGTTTCCAAAGCTATACCTCTATGCCGGTGATTCGCACG  1569TACGCTGAAGATAAAGACATGAACATTGCGTCACCGGTCATCGAACTGCATCAGAAAAAC  1629GTGGGTGGCAATGAACGCATCGCCGGCACCATGTTTCGTTATTTTCGCTTCCCGCGTGAT  1689TTTGAAAATTTCGTGTACCTGTCCCAGGTTCAGCAAGCACTGGCTATTCGTACCGCGGTT  1749GATTATTGGCGCTCACTGAAACCGCATTGTATGGGTACCCTGTACTGGCAACTGAACGAC  1809ACGTGGCCGGTGGCATCCTGGAGCTCTCTGGATTATGGTGGCGGTTGGAAAGCACTGCAC  1869TACGCAGCTCGTCGCTTTTTCCAGCCGGTTGCAGTCAGTGCTATTCCGTCCGCTGATGGC  1929CGTCGCGTCACCTTTAGCATGGTGAACGACACGGCGGAAGATGTGGAAATTGACATGAAT  1989ATCGTTGCGCTGGCGATGGATGGTAACCGTGTTCCGCTGAAATCCGCCAATGGCACCTGC  2049ACGTCAGACAAAGCGGCCACCCTGACGGATATTGACATGGATTCTCTGCCGGATGGTGCG  2109ATTCTGGCCTGGAACTTTATCGCGAGTAATGGCATGACCGGTGAAGGCCATCACGTGCGC  2169GATACGTATAAAGCTCTGGAACTGCAGCCGGCGGGTCTGGAATTTTCGGTGGGTCCGCTG  2229AAAAACGGCCAATTCGAAATTGACGTTACCGCAGCTGGCCTGGCCCTGTTTATCATGCTG  2289GAAGCAGATCAGCCGGGTCGTTACAGCGATAACCTGTTTGACCTGGCGGCCGGCGAAACC  2349CGTCGCATTATCTTCACGCCGAAAGGTGCAGGTCCGCAGCCGCATTTTCGCATTTTCGAT  2409CTGCACACCTGTCAAAGTTCCCCGAATCCGGGCATCGAAACGATGCGTCGCAAAGCG |
| Xylan β-1,4 xylosidase | SlXyn39 | 9 CCGCTGCTGCGTCGCTTCTGTCTGCAAACGGGTCATCTGAGTGCTCTGTTCTTCGGTCTG  69 GTGGTCTCTGTCGCCTTCGGTAGTATCGATAGCCATGCGCAGACCTCTCCGGACAAACCG  129 GTTGCCATCCAAGTCGATCTGACGAAAGACAAAGGCCCGCTGAAACCGATTTGGGCGTGG  189 TTTGGTTATGACGAACCGAACTATACCTACATGAAAGATGGCCGTAAACTGCTGACGGAA  249 ATCAGCCAGCTGTCTAAAGTGCCGGTTAACGTCCGCGTGCACTCACTGCTGGTGACCGGT  309 GACGGTACGGCAGCACTGAAATGGGGCTCGACCAATGCGTATACGGAAGATAAAGCCGGT  369 AACCCGATCTACAACTGGACCATCATCGATAAAATCTTCGACACGTTCATCGAACGTGGC  429 ATGAAACCGATCGCACAGATTGGTTTTATGCCGGAAGCTCTGTCCACCAAACCGCAACCG  489 TATCGCCATTACTGGAAACCGGGCGATAACTATAATGACATCTACACGGGTTGGGCCTAT  549 CCGCCGAAAGATTACAAAAAATGGAGCGAACTGGTGTATCAGTGGGTTAAACACTCTGTC  609 CAGCGTTATGGCCAAAAAGAAGTTGAAAGTTGGTACTGGGAACTGTGGAACGAACCGAAT  669 ATCTCCTATTGGAAAGGTACCACGGAAGAATACATTAAACTGTATGATTACACCGCAGAC  729 GCTGTGAAACGTGCGCTGCCGACCGCCAAAATTGGCGGTCCGGAAGTTACGGGCCCGAAC  789 TGGGACGTCTCTGAAAAATTTTTCCGCGCGTTTATGGATCATGTGGTTAAAGGTACCAAT  849 GCCGTGACGGGCAAAGTTGGTACCCCGATCGATTTTATTACGTTCCACGCAAAAGGTGCT  909 CCGAAAGTCGTGAACGGCGTTGTCCAGATGAATATGGGTACCCAACTGCGTGATATCGAC  969 AAAGGCTTTGCGATTGTGGCCAGTTATCCGTCCCTGAAAAACCTGCCGATTATCATTGGT  1029GAATCAGACCCGGAAGGCTGCGCAGCTTGTTCGGAAGATCTGCATCCGCAGAACGCATAT  1089CGCAATGGTACCATGTATAGCTCTTACACGGCGGCCAGTTTCGCGCGTAAATACGATCTG1149GCAGAAGCCCGCGGCGTCAATCTGGCAGGTGCTGTGACCTGGGGCTTTGAATTCGAAGAT  1209CAGGCATGGTTTCGTGGCTTCCGCGATCTGGCTACGAACGGTGTTGACAAACCGGTCCTG  1269AATGTGTTTCGTATGTTCGGCATGATGCAGGGTAACCGCGTTGCGGTCAATACCGATCTG  1329GCATATAACGCGAAGAAAATTCGTGATGAAAGCGTGCGCGGCGAACCGGACATTAATGCG  1389CTGGCCACCAAAGATGCGCAGAGTGCCTCCGTGATGGTTTGGAACTACCATGATGACAAT  1449GTTCAAGGCCCGGGTTCACCGGTGTCGCTGAAAATCAACGGTCTGACCGCCAAAAAAGTC  1509TGGGTGCAGCACTACCGTATCGATCAGCAATTCTCAAACTCGTACGAAGTTTGGAAGAAA  1569ATGGGCAGCCCGAAAGCACCGACCGCTGAACAGGTGGCAGAACTGGAAAAAGCCGGTCAG  1629CTGCAACTGCTGGCATCTCCGACCTGGGTTACGGTCGAAAAAGGCGTTCTGACCATGCCG  1689TTCCAGCTGCCGCGCCAAGGTGTGAGTCTGGTGAAAGTTTCCTGG |
| Oligosaccharide reducing end xylanase | FjXyn8 | 9 AACGGCTATCTGTCAAAAATTCATCTGCTGCTGCTGATCTTCGCTCTGATTGTTCCGGCA  69 TTCGTCATCTCGCAAAATAAAAAAAAAATTGATCGTGAACTGAAAAAACCGCAGTACCGC  129 AACCTGTTTAAAGAAGCGGGTTACAGTCAAGATGACATCGATAAAAAACTGACCAAAGCC  189 TATTACGACGTGTTTGAAGGCCCGGATAAAGTTTACTTCGAAGAAGGTGATAGTCTGGGC  249 TATGTCTCCGACGTGAAAAATAAAGATGCACGTACGGAGGGTATGAGCTATGGCATGATG  309 GTGGCTGTTCAGTTTAACAAAAAAGATGTCTTCGACCGTCTGTGGCGCTGGTCTGTGAAA  369 TACATGCAGCATCAAGATGGTCCGCGCGAAGGCTATTTTGCATGGAGCGTGAATCCGCAG  429 ACCAAAAAACAAAACTCGGCAGGTAGCGCTTCTGACGGCGAACTGTATTACATTACCTCA  489 CTGCTGTTCGCCTCGAATAAATGGGGTAACGATACGGGCATTAATTATTACAAAGAAGCG  549 CGTCGCATCCTGGACGCCATGTGGAAAAAAGATGGTACCGGCAACATCTACAACATCATC  609 AACACGGAACATAAACAGATCAGCTTTGTCCCGGAAGGCGGTGGCTATAACTGGACCGAC  669 CCGTCTTATCATGTTCCGGCGTTCTACGAAATTTGGGCGCTGTATGCCAAAGATGGTCAC  729 GAACAGTTTTATAAAGAATGCGCGGAAGTGTCACGTAAATTCCTGCATAAAGCCTGTCAC  789 CCGGTTACCGGTCTGACGTCGGATTATACCGAATTTAATGGCGAACCGCACCCGACGCCG  849 TGGCTGCCGCCGGGTTTCCGTTATGACAGCTGGCGCGTGCCGATGAACATCGCAATGGAT  909 TATACCTGGTACGGCAAAGACAAAGAATGGCAGGAAGATTACGCTAAACGTTTTCAAAAT  969 TTCCTGCGCAGTAAAGGCCTGGAAACCTATGAAGACCAGTTTAACCTGGATGGTTCCACG  1029CCGGAATTCATTCTGCAAGCCGGCCCGGTTAAAAAACTGCGCCATAGCATCGGTCTGGTT  1089GGCACCGCAGCAACGGCAAGTCTGGTCAATAAAGACAAAGCTTCCATCGATTTTGTTCAC |
