# Supplementary material for: Characterization of novel glycosyl hydrolases discovered by cell wall glycan directed monoclonal antibody screening and metagenome analysis of maize aerial root mucilage
Source: PLoS One. 2018 Sep 26;13(9):e0204525. doi: 10.1371/journal.pone.0204525 (PMC6157868; doi:10.1371/journal.pone.0204525)
Supplement: S5 Table — The following sequences were downloaded from NCBI GenBank after browsing the CAZy database and were incorporated into the phylogenetic analysis used to generate the trees shown in Fig 4. (DOCX) [file pone.0204525.s010.docx]

| **GH Family** | **NCBI Accession** | **Enzyme Name** | **Taxonomic**  **Classification** |
| --- | --- | --- | --- |
| GH51 | ALK02878.1 | Abf51A | Alicyclobacillus sp. A4 |
| GH51 | ACB54691.1 | Abf | Anoxybacillus kestanbolensis |
| GH51 | ABC55452.1 | AraB | Bacillus pumilus |
| GH51 | CAJ77816.1 | ArfA | Bacillus pumilus |
| GH51 | CAA99595.1 | AbfA | Bacillus subtilis |
| GH51 | CAA99576.1 | Abf2 | Bacillus subtilis |
| GH51 | AAA50393.1 | AsdII | Bacteroides ovatus |
| GH51 | BAF39986.1 | XsA | Bifidobacterium adolescentis ATCC 15703 |
| GH51 | BAF40305.1 | AbfB | Bifidobacterium adolescentis ATCC 15703 |
| GH51 | AAO84266.1 | AbfB | Bifidobacterium longum |
| GH51 | ADT80795.1 | AfuB | Bifidobacterium longum |
| GH51 | AAN24368.1 | AbfA | Bifidobacterium longum NCC2705 |
| GH51 | ACM60204.1 | CbAra51A | Caldicellulosiruptor bescii DSM 6725 |
| GH51 | ABP67153.1 | CsAraf | Caldicellulosiruptor saccharolyticus DSM 8903 |
| GH51 | AEE47435.1 | Abfcelf | Cellulomonas fimi ATCC 484 |
| GH51 | ACE86344.1 | abf51A | Cellvibrio japonicus Ueda107 |
| GH51 | AAN05450.1 | ArfA | Clostridium cellulovorans |
| GH51 | AAC28125.1 | Arf51B | Clostridium stercorarium |
| GH51 | AAC38456.1 | CxAbf1 | Cytophaga xylanolytica |
| GH51 | AAC38457.1 | CxAbf2 | Cytophaga xylanolytica |
| GH51 | EDY06090.1 | LarbF | Geobacillus sp. G11MC16 |
| GH51 | ABM68633.1 | Abf | Geobacillus stearothermophilus |
| GH51 | ALB24935.1 | Abf2 | Geobacillus stearothermophilus |
| GH51 | ALB24934.1 | Abf1 | Geobacillus stearothermophilus |
| GH51 | AAD45520.2 | AbfA | Geobacillus stearothermophilus |
| GH51 | ACE73681.1 | AbfB | Geobacillus stearothermophilus |
| GH51 | ABD48560.1 | AbfA | Geobacillus thermoleovorans |
| GH51 | ADJ95770.1 | Abf1 | Lactobacillus brevis ATCC 14869 |
| GH51 | ADJ95771.1 | Abf2 | Lactobacillus brevis ATCC 14869 |
| GH51 | AFD62907.1 | Abf22-3 | Leuconostoc sp. 22-3 |
| GH51 | ADJ95768.1 | Abf | Oenococcus oeni |
| GH51 | ABZ10760.1 | AbfA | Paenibacillus sp. HanTHS1 |
| GH51 | ABI34800.1 | AbfA | Parageobacillus caldoxylosilyticus |
| GH51 | ADM26764.1 | AbfA | Rhodanobacter ginsenosidimutans |
| GH51 | ABN53749.1 | Araf51A | Ruminiclostridium thermocellum ATCC 27405 |
| GH51 | AEE64774.1 | Ara51a | Ruminococcus albus 8 |
| GH51 | AAA61708.1 | Afase I | Streptomyces lividans |
| GH51 | BAH02662.1 | Afa | Streptomyces sp. I10-1 |
| GH51 | ACY69989.1 | Abf51S9 | Streptomyces sp. S9 |
| GH51 | CAA76421.2 | AbjA | Thermobacillus xylanilyticus |
| GH51 | AAD35369.1 | TxAbf | Thermotoga maritima MSB8 |
| GH51 | ACD60479.1 | XoAf | Xanthomonas oryzae pv. oryzae PXO99A |
| GH51 | A5FF88 | FjArf51 | Flavobacterium johnsoniae ATCC 17061 |
| GH29 | AAO76732.1 | Bt1625 | Bacteroides thetaiotaomicron VPI-5482 |
| GH29 | AAO77299.1 | Bt2192 | Bacteroides thetaiotaomicron VPI-5482 |
| GH29 | AAO78076.1 | Fuc2970 | Bacteroides thetaiotaomicron VPI-5482 |
| GH29 | AAO79241.1 | Bt4136 | Bacteroides thetaiotaomicron VPI-5482 |
| GH29 | BAH80310.1 | BbAfcB | Bifidobacterium bifidum JCM 1254 |
| GH29 | CJ53394.1 | Blon_2336 | Bifidobacterium longum subsp. infantis ATCC 15697 |
| GH29 | ACJ51376.1 | Blon_0248 | Bifidobacterium longum subsp. infantis ATCC 15697 |
| GH29 | ACJ51546.1 | Blon_0426 | Bifidobacterium longum subsp. infantis ATCC 15697 |
| GH29 | ABG83106.1 | Afc2 | Clostridium perfringens ATCC 13124 |
| GH29 | CAQ67115.1 | AlfA | Lactobacillus casei BL23 |
| GH29 | CAQ67877.1 | AlfB | Lactobacillus casei BL23 |
| GH29 | CAQ67984.1 | AlfC | Lactobacillus casei BL23 |
| GH29 | CBM40947.1 | aLfuk1 | Paenibacillus thiaminolyticus |
| GH29 | AAD10477.1 | N/A | Streptomyces sp. |
| GH29 | AEW21393.1 | Fuc1 | Tannerella forsythia 92A2 |
| GH29 | AAD35394.1 | TM0306 | Thermotoga maritima MSB8 |
| GH29 | AIC77302.1 | Mfuc5 | uncultured bacterium |
| GH29 | AIC77298.1 | Mfuc1 | uncultured bacterium |
| GH29 | AIC77299.1 | Mfuc2 | uncultured bacterium |
| GH29 | AIC77301.1 | Mfuc4 | uncultured bacterium |
| GH29 | AIC77303.1 | Mfuc6 | uncultured bacterium |
| GH29 | AIC77304.1 | Mfuc7 | uncultured bacterium |
| GH29 | AAM42160.1 | XCC2888 | Xanthomonas campestris pv. campestris str. ATCC 33913 |
| GH29 | D2QK56 | SlFuc29 | Spirosoma linguale ATCC 33905 LMG 10896 |
| GH39 | BAA95685.1 | XysA | Aeromonas caviae |
| GH39 | BAB04787.1 | BHXyl39 | Bacillus halodurans C-125 |
| GH39 | ADQ03734.1 | CoXylA | Caldicellulosiruptor owensensis OL |
| GH39 | AAA23063.1 | XynB | Caldicellulosiruptor saccharolyticus |
| GH39 | AAB87373.1 | XynD | Caldicellulosiruptor saccharolyticus |
| GH39 | ABP67986.2 | XynD | Caldicellulosiruptor saccharolyticus DSM 8903 |
| GH39 | AAK24328.1 | XynB2 | Caulobacter vicroides CB15 |
| GH39 | CAD48308.1 | BxlA | Clostridium stercorarium |
| GH39 | WP_020755811.1 | N/A | Geobacillus thermocatenulatus |
| GH39 | ABI49941.1 | XynB | Geobacillus stearothermophilus |
| GH39 | AAG05625.1 | PslG | Pseudomonas aeruginosa PAO1 |
| GH39 | AAA27369.1 | XynB | Thermoanaerobacterium saccharolyticum DSM 7060 |
| GH39 | AFK86459.1 | XylB | Thermoanaerobacterium saccharolyticum JW/SL-YS485 |
| GH39 | D2QHX5 | SlXyn39 | Spirosoma linguale (strain ATCC 33905 LMG 10896 |
| GH2 | AAO75565.1 | Man2A | Bacteroides thetaiotaomicron VPI-5482 |
| GH2 | ACM59377.1 | Man2A | Caldicellulosiruptor bescii DSM 6725 |
| GH2 | AAD42775.1 | Man2A | Cellulomonas fimi ATCC 484 |
| GH2 | ACI19676.1 | DtMan | Dictyoglomus thermophilum H-6-12 |
| GH2 | AGX85875.1 | ManB | Paenibacillus polymyxa |
| GH2 | ACN78887.1 | Bma | Streptomyces sp. S27 |
| GH2 | CAD33708.1 | ManB | Thermobifida fusca TM51 |
| GH2 | AAZ54953.1 | ManB | Thermobifida fusca YX |
| GH2 | AAD36691.1 | Man2 | Thermotoga maritima MSB8 |
| GH2 | Q7CZ23 | AfMan2 | Agrobacterium fabrum (strain C58 / ATCC 33970) |
